# *Gardnerella* subgroup dominant microbiomes are associated with divergent cervicovaginal immune responses in a longitudinal cohort of Kenyan women

**DOI:** 10.3389/fimmu.2022.974195

**Published:** 2023-01-16

**Authors:** Elinor Shvartsman, Catia T. Perciani, Meika E. I. Richmond, Justen N. H. Russell, Riley H. Tough, Sarah J. Vancuren, Janet E. Hill, KAVI-ICR, Walter Jaoko, Lyle R. McKinnon, Paul A. Sandstrom, Kelly S. MacDonald

**Affiliations:** ^1^ Department of Medical Microbiology and Infectious Disease, University of Manitoba, Winnipeg, MB, Canada; ^2^ JC Wilt Infectious Diseases Research Centre, Winnipeg, MB, Canada; ^3^ Department of Internal Medicine, University of Manitoba, Winnipeg, MB, Canada; ^4^ Department of Immunology, University of Toronto, Toronto, ON, Canada; ^5^ Department of Veterinary Microbiology, University of Saskatchewan, Saskatoon, SK, Canada; ^6^ Kenyan AIDS Vaccine Initiative-Institute of Clinical Research (KAVI-ICR), University of Nairobi, Nairobi, Kenya; ^7^ Centre for the AIDS Program of Research in South Africa (CAPRISA), Durban, South Africa

**Keywords:** bacterial vaginosis (BV), gardnerella species, vaginal microbiome, HIV susceptibility, mucosal immunity, cpn60, IP-10 (CXCL-10), cytokines

## Abstract

Most cervicovaginal microbiome-immunology studies to date have relied on 16S rDNA microbial profiling which does not resolve the molecular subgroups of *Gardnerella*, believed to be central to the pathogenesis of bacterial vaginosis (BV) and subsequent risk of HIV acquisition. Here we used the *cpn*60 universal target which in addition to other microbial taxa, resolves four *Gardnerella* subgroups, for cervicovaginal microbial profiling in a longitudinal cohort of Kenyan women to examine associations with cellular and soluble markers of inflammation and HIV susceptibility. Participants (N = 41) were sampled, contributing 362 samples for microbiome analysis. All non-*Lactobacillus* dominant microbial communities were associated with high pro-inflammatory cytokine levels. Divergent associations were observed among different *Gardnerella* subgroup dominated communities with respect to the chemokine IP-10. Specifically, *Gardnerella* subgroup A dominant and polymicrobial communities were associated with reduced concentrations of IP-10 in adjusted linear mixed models (p<0.0001), compared to microbial communities dominated by *Lactobacillus* (non-iners) species. However, these associations did not translate to significant differences in the proportion or absolute number of CCR5, HLA-DR and CD38 expressed on cervical CD4^+^ T- cells. These findings suggest that some associations between *Gardnerella* subgroup dominant microbiomes and mucosal immunity differ and are relevant for the study of BV-pathogenesis and understanding the mechanisms of BV-associated HIV risk.

## 1 Introduction

Mucosal host-microbiome interactions in the lower female genital tract (LFGT) play a crucial role in reproductive health. Optimal vaginal microbial communities are thought to be dominated by specific *Lactobacillus* species which confer health benefits *via* production of antimicrobial metabolites including lactic acid, which promote vaginal acidity and possess anti-inflammatory properties (1–3). However*, L. iners* possesses a distinct phenotypic profile in contrast to the other lactobacilli including expression of a pore forming toxin called inerolysin and is associated with transition to more diverse microbial community structures (4, 5). During bacterial vaginosis (BV) the abundance of anaerobic and facultative anaerobic bacteria increases, including *Gardnerella* and *Prevotella* species, accompanied by a reduction or loss of optimal lactobacilli (3). BV can be accompanied by an increase in vaginal pH (pH>4.5) and abnormal malodorous vaginal discharge; however, asymptomatic cases are also common (6–8). Regardless of symptoms, BV and polymicrobial BV-associated communities and taxa have been linked to several sequelae, including increased risk of acquisition and transmission of human immunodeficiency virus (HIV) (9–13). However, the mechanism underlying the BV-HIV link is incompletely understood.

Subclinical LFGT inflammation as measured by increased concentrations of pro-inflammatory cytokines has been associated with BV (14–18). Cervicovaginal inflammation can be measured by increased levels of secreted pro-inflammatory markers including IL-1α, IL-1β, and IL-6 or chemokines including MIP-1α, MIP-1β, and Interferon-γ inducible protein 10 (IP-10) also known as CXCL10. At high concentrations these cytokines can damage epithelial barrier integrity, recruit HIV target cells, and/or induce activation of HIV target cells, thus creating favorable conditions for the establishment of HIV infection. More recently, reduced cervicovaginal IP-10 combined with increased IL-1α and IL-1β have been proposed as immunological biomarkers for BV diagnosis when combined with increases in pH (16, 19). Subclinical genital inflammation has also been linked to increased HIV risk in several cohorts and was shown to reduce the efficacy of a prophylactic topical vaginal gel for HIV prevention in the CAPRISA-004 cohort (20, 21). In addition, high diversity *Lactobacillus*-deficient microbial communities as identified using 16S rDNA sequencing were associated with increased frequency of activated cervical HIV target cells (CD4^+^CCR5^+^CD38^+^HLA-DR^+^ T cells) and increased HIV risk in the FRESH cohort (12, 22). A similar association was noted between BV and number of HIV target cells in a separate cohort (15). However, other studies did not recapitulate these findings (23, 24).

While understanding BV-associated HIV risk can guide HIV prevention efforts, the precise cause of BV has yet to be identified and the condition remains largely enigmatic, resulting in limited interventions that suffer high recurrence rates (25–28). *G. vaginalis*, a gram-variable pleomorphic facultative bacterium has been proposed as the causative agent of BV likely *via* a mechanism involving biofilm formation, however this bacterium is also commonly isolated from healthy individuals (29–33). Next generation sequencing and *cpn*60 barcode sequencing revealed four different *Gardnerella* clades/subgroups, which were recently designated as distinct genomospecies within the *Gardnerella* genus (**Table 1**) (34–38).

**Table 1:** *Gardnerella* spp. Nomenclature.

*cpn*60 subgroup(Jayaprakash et al.)	Clade (Ahmed et al.)	Species and genome species^†^ (Vaneechoutte et al.)	References
**A**	4	*G. leopoldii* (genome sp. 5), *G. swidsinskii* (genome sp. 6), genome sp. 7	(34–37)
**B**	2	*G. piotii* (genome sp. 4), genome spp. 3 and 11	
**C**	1	*G. vaginalis* (genome sp. 1), genome sp. 2	
**D**	3	genome spp. 8, 9, and 10	

^†^Genome spp. 12, and 13 do not fit into the defined subgroups, clades, or species

Phylogenetic and *in-vitro* investigations also suggest differences in ecological and virulence properties among the *Gardnerella* subgroups (38–42). Therefore, these subgroups may possess distinct roles in modulating the pathogenesis of BV and HIV susceptibility in the LFGT. *In vitro* studies of the immune response using different *Gardnerella* isolates (belonging to different subgroups) have provided some discrepant results (43, 44) – highlighting the need to better understand the effects different *Gardnerella* species have on the host immune response *in vivo.* Despite this, most studies to date exploring the cervicovaginal microbiome and mucosal immunology link have relied on microbial profiling using the 16S rDNA universal target, which does not reliably distinguish the different *Gardnerella* subgroups. This in turn has limited our ability to fully elucidate the contributions of these different genomic subgroups to the mucosal immune milieu.

The objective of this study was to determine if *Gardnerella* subgroups segregate into defined microbial communities and examine the associations between these communities and cervicovaginal immune markers that may link specific microbial communities to altered HIV susceptibility. This was done by characterization of the cervicovaginal microbiomes of a longitudinal cohort of Kenyan women using *cpn*60 barcode sequencing and subsequent examination of their associations with cellular and soluble markers of inflammation HIV susceptibility.

## 2 Methods

### 2.1 Study cohort

Kenyan women aged 18-50 (N = 45) at low risk to HIV based on previously outlined criteria (45) and seropositive to varicella zoster virus (VZV) were enrolled in the 48-week KAVI-VZV-001 clinical trial. This trial examined the safety and immunogenicity of the VZV_Oka_ vaccine strain as a potential HIV vaccine vector (ClinicalTrials.gov: NCT02514018). As described by Perciani et al, VZV vaccination did not significantly alter the cervicovaginal immune responses examined in this ancillary study (18). Ethical approvals were granted by the Kenyatta National Hospital/University of Nairobi Ethics and Research Committee, the University of Toronto Research Board and the Kenyan Pharmacy and Poisons Board, with all participants providing informed consent. Participants were scheduled for ~8-10 visits, during which cervicovaginal secretions, vaginal swab samples, and cervical cytobrush samples were collected as previously described (45) (**Supplementary Material Appendix 1**). Of the enrolled participants, 41/45 completed at least 8 visits and were included in this ancillary analysis. All participants were on some form of contraception, with the option to change the contraceptive method throughout the study period (**Supplementary Material Appendix 2**). Throughout the study, participants were able to obtain antimicrobial treatment for concomitant conditions such as BV, and vulvovaginal candidiasis (VVC).

### 2.2 Sample collection and processing

Cervicovaginal secretion samples were collected using a plastic Softcup device (Instead, Evofem Biosciences Inc., San Diego, CA, USA) which was inserted into the vagina for 20 minutes (45). Following collection, secretions were pelleted for 10 minutes at 400 x *g* and treated with a protease inhibitor. Cervicovaginal pellets (CVP) were used for subsequent microbiome profiling, and the remaining cervicovaginal supernatants (CVS) were used for the quantification of cytokine concentrations. To prevent potential interferences caused by blood-derived products in cervicovaginal samples, participants undergoing menses or spotting during the scheduled collection visit were rescheduled for sample collection, typically within a week from the original scheduled visit. Cellular data was collected using a cervical cytobrush (Digene, QIAGEN Inc., Toronto, ON, Canada) which was inserted into the cervical canal and rotated once. Vaginal swabs were collected at each visit for the diagnosis of BV using the Nugent criteria (Nugent-BV) and VVC using microscopy as previously described (45, 46). HSV-2 seropositivity was measured only at enrollment (18).

### 2.3 Microbiome analysis

CVPs were resuspended in 1 mL phosphate buffer saline (PBS, pH 7.5). Then, microbial DNA was extracted using the DNeasy Blood and Tissue Kit protocol (QIAGEN Inc., Toronto, ON, Canada) modified to include a lysozyme and mutanolysin pre-treatment step to enhance bacterial cell wall lysis as previously described (47). *cpn*60 amplification was done using the M729, M730, M1612, and M1613 primer combinations as previously described (47, 48). Following index-PCR, products were cleaned-up using the AMPure XP beads (Beckman Coulter Canada, Mississauga, ON, Canada) per manufacturer’s protocol. Following this, amplicon concentrations were normalized to 4 nM and pooled in preparation for sequencing using the NextSeq 500/550 Mid Output Kit v2 (300 Cycles) (Illumina Canada Inc., Toronto, ON, Canada). Sequencing was performed by the DNA Core facility at the National Microbiology Laboratory in Winnipeg, Canada using a single 1 x 299 run on the Illumina NextSeq machine with sequencing from the 5’ end of the *cpn*60 amplicon target. Reads were then demultiplexed and processed using the QIIME2 analysis pipeline (49). Amplicon sequence variant (ASV) assignment was done using DADA2 (50). Reads greater than or equal to 250 base pairs in size were included in subsequent analysis. ASVs were assigned an identity according to their nearest neighbor reference sequence as determined by the Smith-Waterman basic local alignment search tool algorithm (watered-BLAST) which compared ASV sequences to the chaperonin database (www.cpndb.ca). A final ASV table was generated, where only ASVs with equal to or greater than 55% identity to a cpnDB reference sequence were used for analysis (51). To obtain initial microbial clusters or community state types (CST) we performed cluster analysis using unsupervised hierarchical clustering with Euclidean distance matrix and Ward.D linkage method using normalized proportions of the raw reads. The number of initial clusters was determined using the ClValid package on RStudio (version 3.5.1) with clustering input of 2:16 clusters. Following hierarchical clustering, non-iners *Lactobacillus* dominant CSTs were further aggregated into a single CST to serve as the reference group in subsequent analysis; this was to account for the ambiguous role that *L. iners* plays in the LFGT (4, 5). Two samples which initially clustered into the polymicrobial community but were comprised of 100% non-iners *Lactobacillus* species were added into the corresponding *Lactobacillus* dominant CSTs. Shannon alpha diversity indices (measuring within sample diversity) were calculated using the phyloseq package (version 1.24.2) on RStudio (version 3.5.1).

### 2.4 Cytokine analysis

CVS were used for the detection of fourteen cytokines (TNF-α, IL-1β, IL-1α, IFN-γ, IL-8, IL-6, IP-10, MIP-3α, MIP-1α, MIP-1β, GM-CSF, IL-10, IL-4, and IL-17A) using the electrochemiluminescence U-PLEX assay (Meso Scale Discovery, Rockville, MD, USA) following manufacturer’s instructions. Samples were run in duplicate, and the average value was used for subsequent analysis. Measurements below the lower limit of detection (LLOD) were given the LLOD for the specific cytokine as determined by the standard curve. Cytokine measurements above the upper limit of detection were assigned the highest value calculated beyond the fit curve for the specific cytokine measured.

### 2.5 Flow cytometry

Cervical cytobrush samples were collected and used for the quantification of cervical T-cells expressing the following markers: CD4, CCR5, CD38, HLA-DR, CD69, and Ki67, or the integrin markers αEβ7, α4β7, and α4β1. Staining using the LIVE/DEAD fixable Far Red Dead Cell Stain Kit (Invitrogen, Carlsbad, CA, USA) was performed as soon as samples were collected. Viable cells were then labelled with the following monoclonal antibodies as previously described by Perciani et al. (18, 52): CD3 APCeFluor780 (clone: SK7), HLA-DR FITC (L243), CD49d PE (9F10), Ki67 PE-Cyanine7 (20Raj1) from eBioscience; CD4 BV510 (clone: SK3), CD38 PE-CF594 (clone: HIT2), CCR5/CD195 BV421 (clone: 2D7) from BD Horizon; CD69 (clone: FN50) and β7 PECy5 (clone: FIB504) from BD Pharmingen; and the antibodies against CD103 (clone: Ber-ACT8) from BioLegend. Rainbow beads (Spherotech, Lake Forest, IL, USA) were used for instrument calibration to ensure consistency in data collection throughout the study period. Flow cytometry of the samples was performed on a BD LSR II flow cytometer using the DiVa software package (BD Biosciences, San Jose, CA, USA). Cellular data processing was conducted using FlowJo (TreeStar Inc.). The gating strategy for the cellular markers used in this study has been previously published (18, 52). Cellular samples were not acquired on week 36 of the study, and downstream analysis also excluded samples that did not pass the flow cytometer’s quality control cutoff. For analysis of relative frequencies, CD4 marker expression was analyzed in relation to the total of CD3 expressing cells (T-cells); CCR5, CD69, CD38, HLA-DR, Ki67, αEβ7, α4β7, α4β1, and co-expression of CD38 and HLA-DR were analyzed in relation to the total CD4 expressing T-cells. For total cell count associations, raw counts were log2-transformed to reduce skewness.

### 2.6 Statistical analysis

To reduce the dimensionality of the cytokine dataset, principal component analysis (PCA) was performed using IBM SPSS Statistics (Version 23) as previously described (18). Briefly, sampling adequacy was measured by the Kaiser-Meyer-Olkin test, and Bartlett’s test of sphericity using all log10-normalized fourteen cytokine measurements collected throughout all the study visits. Component extraction was performed on the correlation matrix based on Eigen values greater than 1 with direct oblimin (oblique) rotation (delta = 0). Bartlett’s method was then used to calculate principal component scores which were subsequently used for statistical analysis. To account for the hierarchical structure of our longitudinal data in the analysis, linear mixed models (LMM) were used to examine the associations between microbial communities and each of the principal components (PC). LMMs were also run separately for each of the fourteen log10-transformed cytokines and the log2-transformed cell counts. LMMs were fitted with aggregated microbial groupings as fixed effects predictors using *Lactobacillus* (non-iners) dominant microbiomes as the reference group. Each model was fitted with a random intercept at the participant level to account for inter-individual variation in the outcome. LMMs were derived using restricted maximum likelihood (REML) estimations using Statistical Analysis Software (SAS). Satterthwaite method for approximation of the degrees of freedom was employed as this method was shown to be fairly robust to type-1 errors regardless of sample size when using REML estimations in LMMs (53). To analyze cellular data relative frequencies (proportions), we used LMM diagnostics to check the suitability of this dataset for LMM modelling, alongside running a beta regression mixed model using PROC GLIMMIX on SAS. Our beta regression models (not shown) mostly agreed with the results from our LMMs although some of the models ran into convergence issues which could not be resolved given the iterative nature of beta regression. Thus, we decided to proceed with LMM for analysis using PROC MIXED to model the associations with microbial groupings. Inferences regarding statistical significance all used alpha = 0.05. Models were adjusted for age, VVC, use of antibiotics (categorized as either BV approved antibiotics such as tinidazole and clindamycin or other, broad-spectrum antibiotics), use of antifungals, and use of hormonal contraception (with non-hormonal intrauterine device [IUD] used as reference), and HSV-2 seropositivity at baseline on the basis that these factors can influence either the microbiome or the mucosal immune milieu. Our adjusted and unadjusted models produced similar outputs, and only adjusted p-values are reported here. Figures in this manuscript were generated using GraphPad Prism 9 and RStudio (version 2021.09.2 + 382).

## 3 Results

### 3.1 Cervicovaginal microbial communities and temporal demographics in the KAVI-VZV-001 cohort

**Figure 1:**
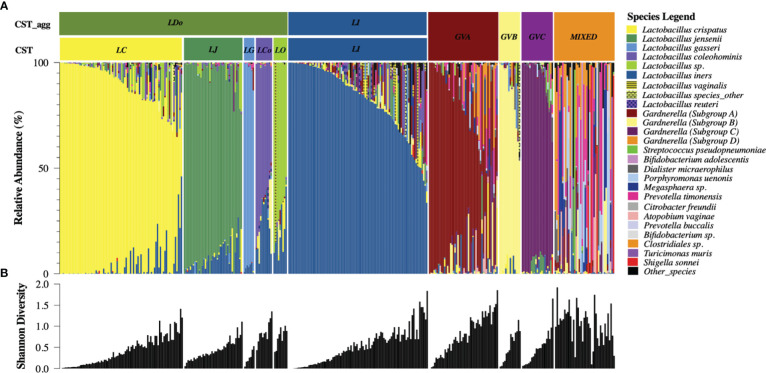
*cpn*60 microbial profiling of cervicovaginal samples reveals six aggregated microbial community structures with variable alpha diversity **(A)** Cervicovaginal microbial profiles obtained using *cpn*60 sequencing from 362 cervicovaginal secretion samples collected from 41 study participants. Profiles were organized by descending relative abundance and aggregated into six functional microbial clusters (CST_agg). The 25 most prevalent microbial taxa are shown with the rest of the species grouped as “other_species” and “Lactobacillus_other_species”. CST – refers to the raw community state type as determined using hierarchical clustering, CST_agg - aggregated CSTs where *Lactobacillus* (non-iners) dominated communities were further aggregated for analysis consistent with current literature. The six aggregated CSTs determined by the dominating microbial taxa are: LDo - *Lactobacillus* (non-iners) dominance, LI - *L. iners* dominance, GVA -*Gardnerella* subgroup A dominance, GVB - *Gardnerella* subgroup B dominance, GVC - *Gardnerella* subgroup C dominance, MIXED – polymicrobial community structure. **(B)** Shannon diversity measure of community alpha diversity shown per corresponding sample.

**Figure 2:**
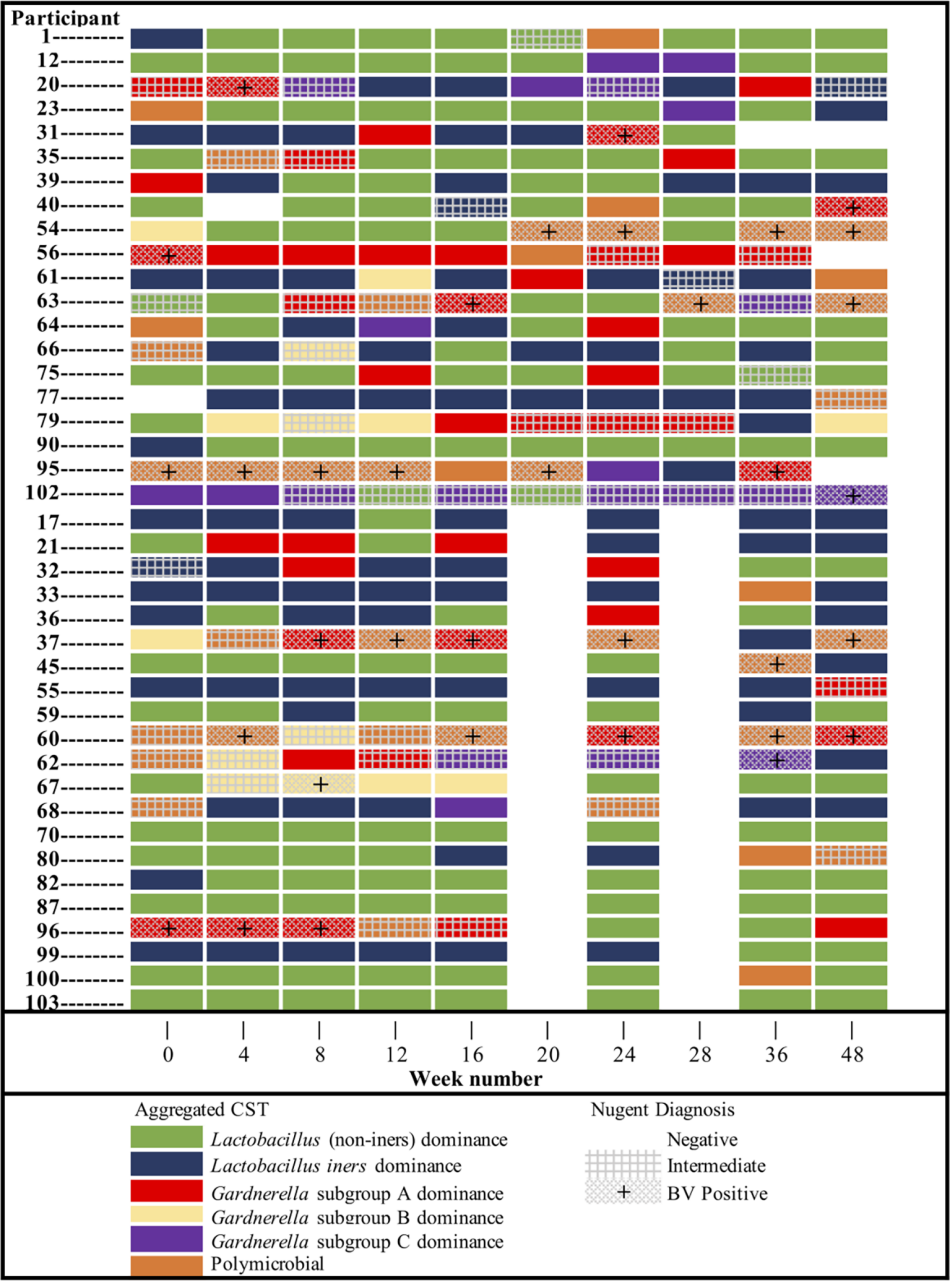
Cervicovaginal microbial community (CST_agg) dynamics of KAVI-VZV-001 trial participants over all study visits throughout the 48-week trial and the corresponding Nugent diagnosis status per visit. Participants are presented divided by sample collection groups as per the main study protocol.

Temporal demographics of KAVI-VZV-001 cohort participants are described in **Supplementary Material Appendix 2**. Ten microbial clusters (CSTs) were resolved using unsupervised hierarchical clustering of 362 cervicovaginal microbial profiles obtained from 41 participants (**Figure 1A**). These clusters included six lactobacilli dominant microbial CSTs with high relative abundances of *L. crispatus* (LC), *L. jensenii* (LJ), *L. gasseri* (LG), *L. coleohominis* (LCo), and a community dominated by other *Lactobacillus* species (LO); these were further aggregated into a single cluster of ‘non-iners’ lactobacilli dominant communities (LDo), and a separate community dominated by *L. iners* (LI). In addition, we identified four non-*Lactobacillus* dominant CSTs including three CSTs dominated by one of the following *Gardnerella* subgroups – subgroup A (GVA), subgroup B (GVB), subgroup C (GVC), and one polymicrobial community (MIXED). A *Gardnerella* subgroup D dominant community was not observed, as this taxon was mostly found in polymicrobial (MIXED CST) communities (**Figure 1A**). Alpha diversity was used as a measure of within sample diversity and was shown to be variable both within and between each CST resolved using *cpn*60 (**Figure 1B**). We also examined the temporal changes in the participants’ cervicovaginal microbiome over the 48-week study period, observing that only ~19.5% (8/41) of participants retained a *Lactobacillus* dominant microbiome throughout all collection visits (either LDo or LI), with majority of samples being non-*Lactobacillus* dominant at least once during a sample collection visit (**Figure 2**, **Supplementary Material Appendix 2**).

### 3.2 Associations of cervicovaginal microbial communities with mucosal cytokines

**Figure 3:**
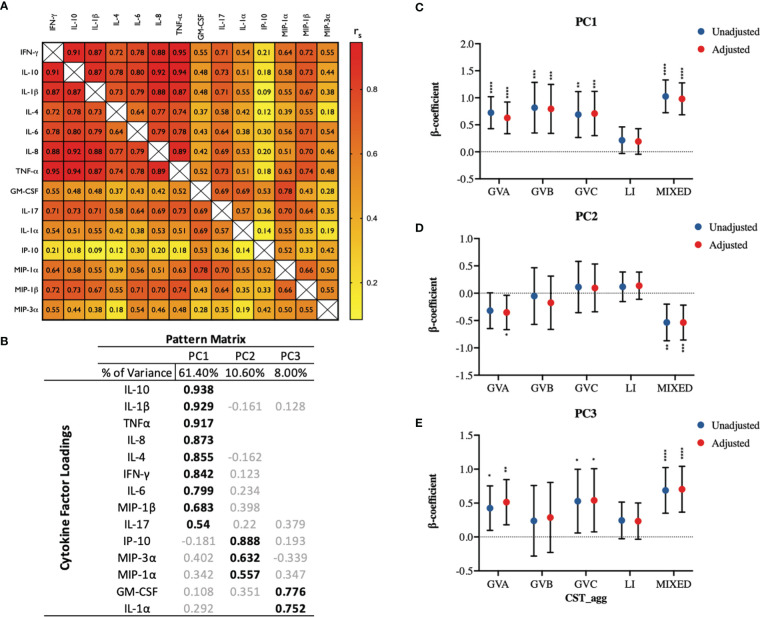
Principal component analysis identifies three principal components of fourteen measured cervicovaginal cytokines which exhibit divergent associations with cervicovaginal microbiome structures (CST_agg). **(A)** Spearman correlation matrix showing correlations among all fourteen measured cytokines. **(B)** PCA pattern matrix (with oblique rotation) showing percent of the variance accounted for by each principal component (PC) and the cytokine loading on each PC. Linear mixed models examining microbial grouping associations with **(C)** PC1, **(D)** PC2, and **(E)** PC3 scores. The β-coefficients represent the mean change in specific PC scores associated with a specific microbiome type (CST_agg) compared to *Lactobacillus* (non-iners) dominant microbial communities. Blue symbols represent unadjusted models only utilizing microbial groupings as predictors. Red symbols represent models following multivariable adjustment for hormonal contraceptive type, concomitant vulvovaginal candidiasis, age, HSV -2 seropositivity at baseline, use of BV antibiotics, use of other antibiotics, and use of antifungals prior to sample collection. The error bars represent the 95% confidence intervals associated with the estimates from the linear mixed models. *P≤ 0.05, **P ≤ 0.01, ***P ≤ 0.001, ****P ≤ 0.0001.

Next, we examined how genital inflammatory and chemotactic cytokine concentrations in cervicovaginal microbial communities dominated by *L. iners* and non-*Lactobacillus* species, compared to *Lactobacillus* ‘non-iners’ dominant communities. Cytokine principal component analysis produced three main PCs based on our specified cut-off criteria, with PC1 explaining a substantial proportion of the variability (61.4%), followed by PC2 (10.6%) and then PC3 (8.0%; **Figures 3A, B**). PC1 was strongly loaded by nine cytokines including the pro-inflammatory cytokine IL-1β, and the immune-modulatory cytokine IL-10. GVA (p<0.0001), GVB (p=0.0006), GVC (p=0.0007) and MIXED (p<0.0001) microbial communities were all associated with significant increases in PC1 scores compared to ‘non-iners’ *Lactobacillus* communities in the adjusted models (**Figure 3C**). LI trended toward an increase in PC1 scores in adjusted models (p=0.1118, **Figure 3C**). PC2 was predominantly associated with the chemokine IP-10 and to a lesser extent the chemokines MIP-3α and MIP-1α (**Figure 3B**). Both GVA (p=0.0278) and MIXED (p=0.001) microbial communities were associated with reduced PC2 scores in the adjusted models (**Figure 3D**). PC3 was strongly influenced by both the growth factor GM-CSF and the pro-inflammatory cytokine IL-1α (**Figure 3B**). Compared to LDo microbial communities, GVA (p=0.0026), GVC (p=0.0232), and MIXED (p<0.0001) microbial communities were all associated with increased PC3 scores in the adjusted models (**Figure 3E**). PC3 scores were higher in the context of LI, but this was not significant (p=0.0866).

**Figure 4:**
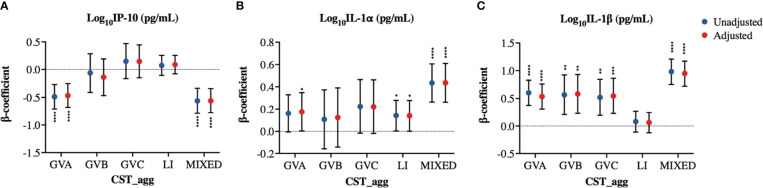
Cervicovaginal microbiome associations with log10-normalized BV-associated cytokines. Linear mixed model estimates for microbiome associations with **(A)** IP-10, **(B)** IL-1α, and **(C)** IL-1β log10-transformed concentrations in pg/mL. β-coefficients represent the estimated mean change in cytokine concentrations associated with a specific CST_agg when using *Lactobacillus* (non-iners) dominant CST_agg as reference. Blue symbols represent unadjusted models only utilizing microbial groupings as predictors. Red symbols represent models following multivariable adjustment for hormonal contraceptive type, concomitant vulvovaginal candidiasis, age, HSV -2 seropositivity at baseline, use of BV antibiotics, use of other antibiotics, and use of antifungals prior to sample collection. The error bars represent the 95% confidence intervals associated with the estimates from the linear mixed models. *P≤0.05, **P≤0.01, ***P≤0.001, ****P≤0.0001.

Associations of cytokine PCs with microbial CSTs were widely recapitulated in analyses using individual log10-transformed cytokines as outcomes (**Figures 4A–C**, **Supplementary Material Appendices 3, 4**). Due to their proposed role as biomarkers of BV, we performed a closer examination of the associations between microbial groupings and the cytokines IL-1α, IL-1β, and IP-10. GVA was associated with increased IL-1α (p=0.0454) and IL-1β (p<0.0001) and with reduced IP-10 (p<0.0001; **Figures 4A–C**). MIXED microbial communities were also associated with reduced IP-10 levels (p<0.0001), and increased IL-1β and IL-1α (both p<0.0001; **Figures 4A–C**). GVB was only associated with significant increases in IL-1β (p=0.0012; **Figure 4C**). GVC was associated with increased IL-1β (p=0.0008), and a trend to increased IL-1α (p=0.0707) (**Figures 4A–C**). Interestingly, LI microbial communities were associated with increased IL-1α levels (p=0.0469; **Figure 4C**). The association of GVA and MIXED microbial communities with reduced IP-10 levels could not be explained by their associations with the IP-10 inducer, IFN-γ (**Supplementary Material Appendix 3**).

### 3.3 Longitudinal patterns in microbial structures and cytokine principal components

**Figure 5:**
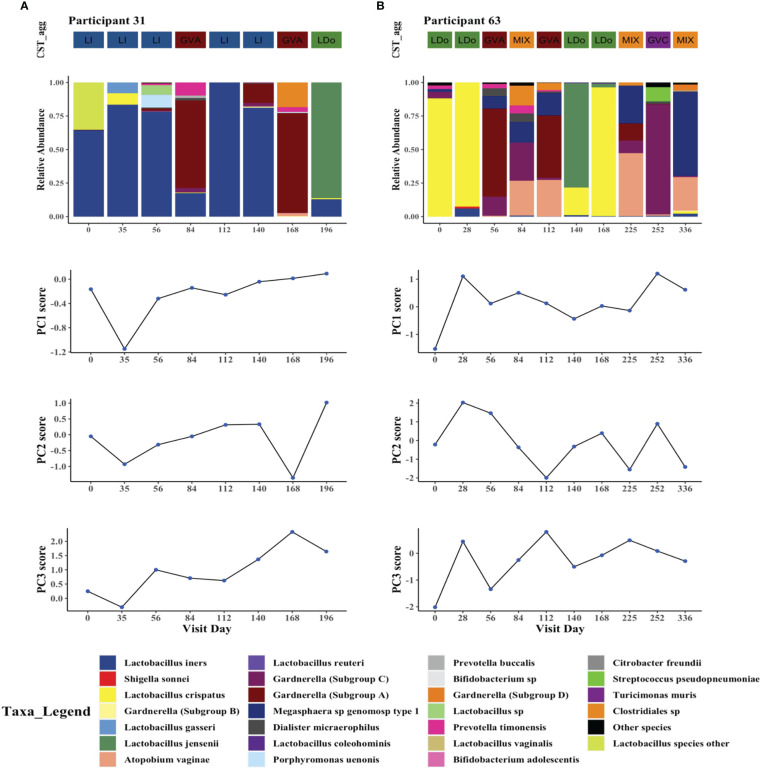
Intraindividual temporal microbiome changes correlate with changes in cervicovaginal cytokines in two study participants. Longitudinal changes in cervicovaginal microbiome profiles and concomitant changes in cytokine principal components PC1, PC2, and PC3 in **(A)** Participant 31, and **(B)** Participant 63. The 25 most prevalent microbial taxa are shown with the rest of the species grouped as “other_species” and “Lactobacillus_other_species”.

We next examined whether transitions in microbial structures over time closely parallel changes in cytokine principal components within individuals. To do this we visualized changes in cervicovaginal microbial profiles over time that coincided with changes in PC1, PC2, and PC3 scores (**Figuress 5A, B**, **Supplementary Material Appendix 5**). In general, CST shifts were associated with subsequent changes in PC scores, with some inter-participant heterogeneity and occasional discrepancies (**Figures 5A, B**, **Supplementary Material Appendix 5**). As examples, participants 31 and 63, who had at least one visit with a LDo CST and at least one visit dominated by non-*Lactobacillus* taxa, mostly recapitulated the findings from our linear mixed models (**Figures 5A, B**).

### 3.4 Associations of cervicovaginal microbial communities with cervical CD4^+^ T-cells and T-cell subsets associated with HIV susceptibility

**Figure 6:**
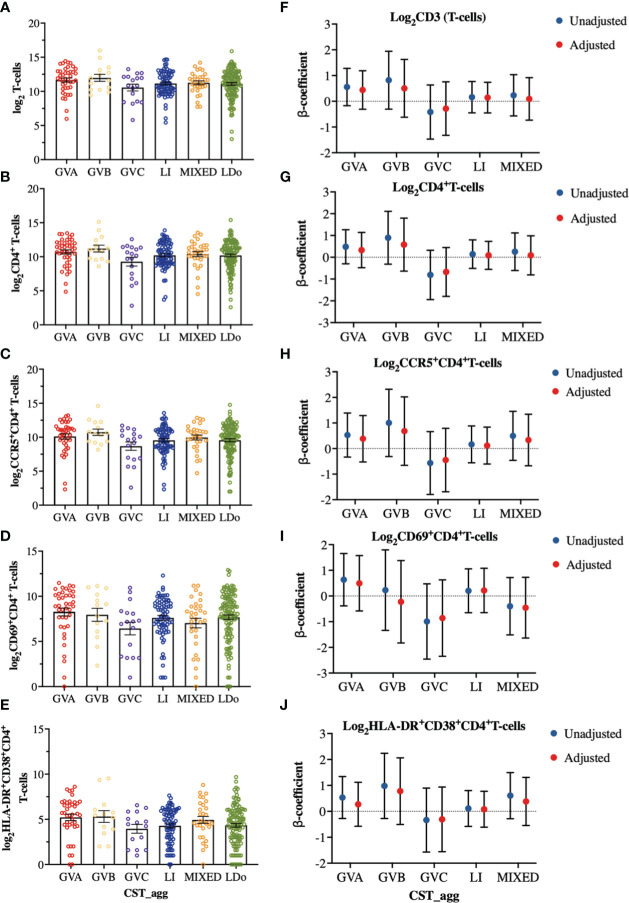
Comparison of cervical T-cell and subsets cell counts among the different cervicovaginal microbial groupings. log2-transformed cervical counts of **(A)** CD3 (T-cells), **(B)** CD4^+^ T-cells **(C)** CCR5^+^CD4^+^ T-cells, **(D)** CD69^+^CD4^+^ T-cells and **(E)** HLA-DR^+^CD38^+^CD4^+^ T-cells. Linear mixed model analysis of the association between specific cervicovaginal microbial communities (CST_agg) and log2-transformed cell counts of **(F)** CD3 (T-cells), **(G)** CD4^+^ T-cells **(H)** CCR5^+^CD4^+^ T-cells, **(I)** CD69^+^CD4^+^ T-cells and **(J)** HLA-DR^+^CD38^+^CD4^+^ T-cells. β-coefficients represent the mean change in log2-transformed cell count associated with a specific microbiome (CST_agg) when compared to *Lactobacillus* (non_iners) dominant (LDo) microbial communities. Blue symbols represent unadjusted models only utilizing microbial groupings as predictors. Red symbols represent models following multivariable adjustment for hormonal contraceptive type, HSV- 2 seropositivity at baseline, concomitant vulvovaginal candidiasis, age, use of BV antibiotics, use of other antibiotics, and use of antifungals prior to sample collection. The error bars in the raw cell count panels represent the standard error of the mean. The error bars from the linear mixed models represent the 95% confidence intervals associated with the estimates.

**Figure 7:**
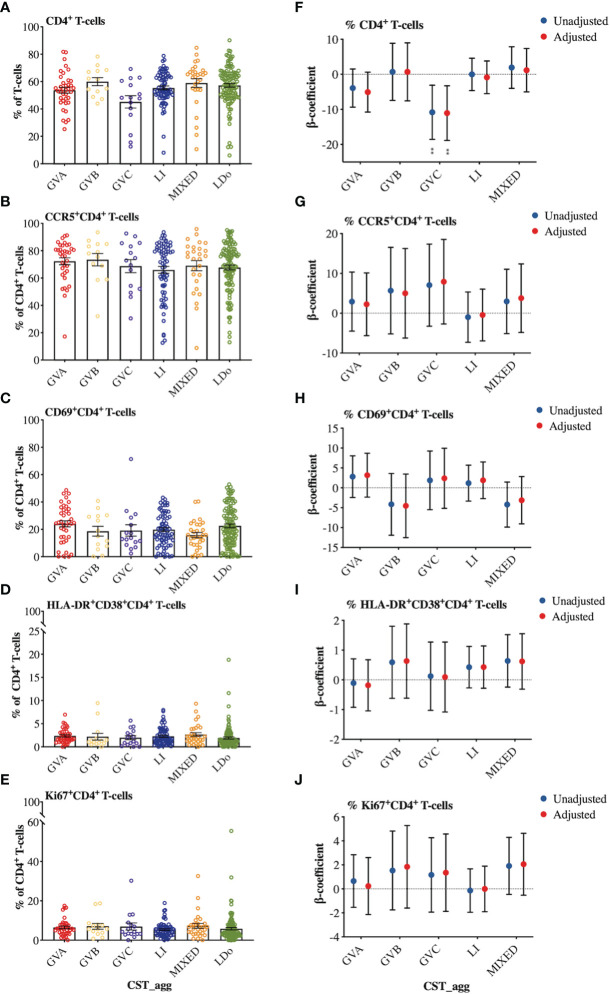
Comparison of cellular marker expression on cervical T-lymphocytes among the different cervicovaginal microbial groupings. Raw relative frequencies of **(A)** CD4^+^ T-cells expressed as percent of the total T-cells (CD3^+^) and the markers **(B)** CCR5 **(C)** CD69 **(D)** HLA-DR and CD38 **(E)** Ki67 expressed as percent of total CD4^+^ T-cells. Linear mixed model analysis of the association between specific cervicovaginal microbial communities (CST_agg) and relative frequencies of **(F)** CD4^+^ T-cells expressed as percent of total T-cells (CD3^+^) and the markers **(G)** CCR5 **(H)** CD69 **(I)** HLA-DR and CD38 **(J)** Ki67 expressed as percent of total CD4^+^ T-cells. β-coefficients represent the mean change in relative frequency of a specific cellular marker that is associated with a specific microbiome (CST_agg) when compared to *Lactobacillus* (non_iners) dominant (LDo) microbial communities. Blue symbols represent unadjusted models only utilizing microbial groupings as predictors. Red symbols represent models following multivariable adjustment for hormonal contraceptive type, HSV- 2 seropositivity at baseline, concomitant vulvovaginal candidiasis, age, use of BV antibiotics, use of other antibiotics, and use of antifungals prior to sample collection. The error bars in the raw cell count panels represent the standard error of the mean. The error bars from the linear mixed models represent the 95% confidence intervals associated with the estimates from the linear mixed models. **P ≤ 0.01.

**Figure 8:**
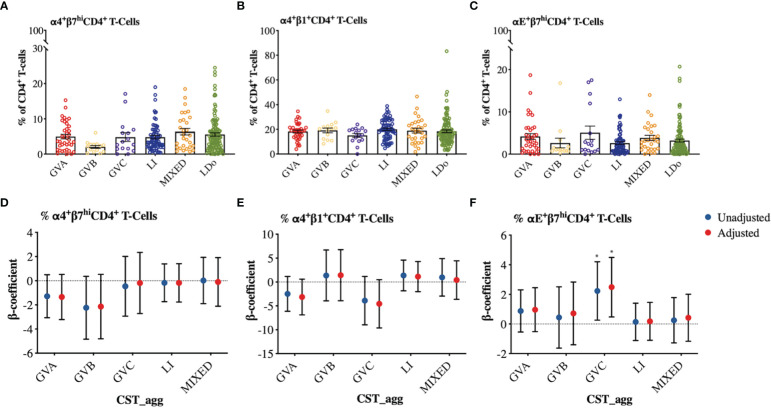
Comparison of integrin expression patterns among different cervicovaginal microbial groupings. Raw relative frequencies of **(A)** α4^+^β7^hi^ **(B)** α4^+^β1^+^ **(C)** αE^+^β7^hi^ cells expressed as percent of total CD4^+^ T-cells shown per CST_agg. Linear mixed model analysis examining the association between cervicovaginal microbial groupings and **(D)** α4^+^β7^hi^ **(E)** α4^+^β1^+^ **(F)** αE^+^β7^hi^ expressed as percent of total CD4^+^ T-cells. β-coefficients represent the mean change in relative frequency of a specific cellular marker that is associated with a specific microbiome structure (CST_agg) compared to *Lactobacillus* (non_iners) dominant microbial communities. Blue symbols represent unadjusted models only utilizing microbial groupings as predictors. Red symbols represent models following multivariable adjustment for hormonal contraceptive type, HSV-2 seropositivity at baseline, concomitant vulvovaginal candidiasis, age, use of BV antibiotics, use of other antibiotics, and use of antifungals prior to sample collection. The error bars in the raw cell count panels represent the standard error of the mean. The error bars from the linear mixed models represent the 95% confidence intervals associated with the estimates from the linear mixed models. *P ≤ 0.05.

We next examined how cervicovaginal microbial community structures are associated with cervical T-cell counts and subsets such as activated CD4^+^ T-cells and those expressing HIV-co-receptors or integrin markers. No statistically significant associations were found between microbial communities and log2-transformed cervical T-cells and CD4^+^ T-cell subset counts (**Figure 6**, **Supplementary Material Appendices 6, 7**). In contrast, GVC was associated with reduced CD4 expression on T cells, relative to LDo communities (p=0.0055), with GVA showing a similar trend (p=0.08; **Figures 7A, F**). GVA, GVB, GVC, LI, and MIXED microbial communities were not associated with the expression of CCR5, CD38, CD69, HLA-DR, Ki67, and CD38/HLA-DR co-expression on CD4^+^ T-cells compared to *Lactobacillus* (non-iners) dominant (LDo) microbial communities (**Figures 7B–E, G–J**, **Supplementary Material Appendix 8**). GVA, GBV, GVC, MIXED, and LI communities did not exhibit any significant associations with the integrin markers α4β1 and α4β7 relative to LDo communities, although GVA (p=0.0992) and GVC (p=0.0766) microbial communities trended towards lower α4β1 expression on CD4^+^ T-cells (**Figures 8A–F**). GVC microbial communities were associated with increased relative frequency of αE^+^β7^hi+^CD4^+^ T-cells compared to LDo microbial communities in our adjusted model (p=0.0155, **Figure 8F**).

## 4 Discussion

Here *cpn*60 microbial profiling of cervicovaginal specimens was performed to describe specific microbial structures associated with changes in the local immune milieu in a low-risk longitudinal cohort. Studies to date examining mucosal immunity and microbiome/BV associations have been (with few exceptions) cross-sectional and in populations either pregnant or at high risk for sexually transmitted infections and given the impact of those conditions on mucosal immunity may not be generalizable to the general population (12, 22, 24, 54, 55). Furthermore, none of these studies employed *cpn*60 barcoding or whole genome sequencing so they were unable to examine the contributions of different *Gardnerella* subgroups to mucosal immunity. Utilizing the *cpn*60 universal target allowed us to decipher three additional microbial community structures dominated by different subgroups of *Gardnerella* to address some of these knowledge gaps. Our findings reveal that microbiome associations with the chemokine IP-10 (and PC2 scores), diverge depending on the type of *Gardnerella* subgroup dominance. These chemokine differences did not translate to significant differences in cervical HIV target cell counts or immune activation of cervical CD4^+^ T-cells. It was also observed that all non-lactobacilli dominant microbiomes were generally associated with an increase in pro-inflammatory cytokines.

BV is predominantly a biofilm condition, with some *Gardnerella* species hypothesized to be more likely to establish biofilms and produce specific virulence factors (32, 40). In this study GVA and polymicrobial communities (but not the other *Gardnerella* subgroup dominant communities) exhibited a strong negative association with IP-10. This suggests that the reported IP-10 suppression associated with BV (16, 18, 19) could be mediated by some *Gardnerella* subgroups and anaerobic taxa but not others. The ability of some *Gardnerella* species to induce this response could confer an ecological advantage to allow persistence within the vaginal niche. Relevant to this – *Gardnerella* subgroup A appear to be the most common of the four *Gardnerella* subgroups in vaginal specimens regardless of BV status (56, 57), including in this study. In addition, IP-10 and related chemokines have been shown to possess direct antimicrobial effects on some gram-positive and gram-negative bacteria (58, 59). The IP-10 receptor - CXCR3, is predominantly expressed on activated T-cell subsets, especially CD8^+^ memory T-cells as well as on innate lymphocytes including natural killer cells and gamma delta T-cells (60–62). The reduction in IP-10 levels during BV could thus potentially explain the reported lower ectocervical CD8^+^ T-cell levels during persistent BV (15), and BV-associated reduced levels of endocervical γδ 1 T cells (63). These understudied cell populations may play protective roles in the defense against bacterial biofilms including those associated with BV (64). We hypothesize that the ability of BV-associated *Gardnerella* species and related BV-associated bacteria to persist and establish BV is therefore dependent at least in part on the ability to suppress this IP-10 response. Understanding the significance and mechanism of mucosal IP-10 suppression to BV and associated sequela could be the focus of future investigations and may be important for the design of future interventions.

These findings are supported by previously published *in-vitro* investigations. Garcia et al, reported a similar IP-10 reduction in an organotypic vaginal-ectocervical tissue following challenge with a *Gardnerella* subgroup A isolate (44). Another study found that *in-vitro* stimulation of endocervical cells with a subgroup C *Gardnerella* isolate significantly increased IP-10 levels, although no significant associations were observed when vaginal and ectocervical epithelial cells were used (43). Relevant to this, reduced IP-10 levels have been reported during Nugent-BV (16, 18, 19) and with BV persistence following antibiotic treatment (28, 65).

Non-*Lactobacillus* dominant microbial communities were associated with a general increase in mucosal markers of inflammation. This finding is consistent with previous studies using 16S rDNA based microbial profiling suggesting *Gardnerella* dominance and/or polymicrobial community structures are associated with increased mucosal pro-inflammatory cytokines (12, 22, 24, 54, 55). Increased levels of IL-1α and IL-1β as well as other pro-inflammatory markers have been also reported in vaginal samples from women diagnosed with BV by Nugent (score of 7-10) and/or Amsel criteria (presence of >2 clinical signs) (16–19, 65).

Despite the microbiome and cytokine associations noted in this study, no significant associations were found between microbial community structure and T-cell markers of adaptive immune activation including HLA-DR and CD38 co-expression, Ki67, and CD69 expression on CD4^+^ T-cells. We also did not identify any significant associations between microbial communities and cervical T-cell counts and/or activated subsets in this cohort. Findings from previous studies regarding microbiome associations with cellular immune activation have also been inconclusive and differed based on the method used for analysis. Gosmann and colleagues found using a 16S rDNA microbial profiling approach, that microbial cervicotypes dominated by *G. vaginalis* and polymicrobial communities were associated with a 9X and a 17X increase, respectively, in the number of CCR5^+^CD38^+^HLA-DR^+^CD4^+^ T-cells, although no information was provided regarding the relative expression of these markers (12). A different study investigating the association of Nugent-BV and cervical cell counts found that women with BV had lower counts of T-cells and HLA-DR expression, but higher levels of CCR5 expression (15). However, in contrast to these studies and in agreement with ours, a study by Lennard and colleagues using a 16S rDNA microbial profiling, also did not identify significant differences in vaginal microbiome associations with relative frequencies of these immune cells (24). Our findings thus support the hypothesis that non-*Lactobacillus* dominant microbial communities promote mucosal inflammation which may in turn increase HIV susceptibility but are not necessarily associated with increased HIV target cells in this low-risk cohort. Based on the divergent IP-10 findings, it is possible other immune cells or effector functions not examined by us such as CD8^+^ T-cell responses are affected by the microbiome to a greater extent which may also explain the widely reported BV and HIV association.

Our study has several limitations. We were unable to perform quantitative analysis at a genomic level to determine the bacterial load in the study samples, so this study is limited to compositional microbiome data. Future investigations combining compositional sequencing approaches with quantitative techniques should provide greater appreciation of the intricate relationship of the cervicovaginal microbiome and the mucosal immune milieu. Our conclusions regarding cellular markers and cytokine associations with microbial structure are limited by the relatively small sample size of independent participants, and a limited number of samples identified as possessing GVC and GVB CSTs. In addition, the flow cytometry panel was limited to an examination of only CD4^+^ T-cells and selective activation and integrin markers – limiting our ability to examine associations with other cell populations due to instrumentation and cell numbers.

In conclusion, this study highlights the importance of *Gardnerella* heterogeneity in the context of mucosal immunology, and comments on cervicovaginal microbial associations with mucosal immunity in a low-risk cohort. While *Gardnerella* dominant and polymicrobial microbiome associations with mucosal inflammation were mostly congruent, striking differences were noted in their associations with the proposed immunological BV-biomarker, IP-10. Future investigations could comment on whether this IP-10 reduction associated with some microbiomes confers ecological advantage within the vaginal ecosystem and its relevance to BV pathogenesis. Additionally, we did not identify differences in CD4^+^ T-cells among the microbial structures, suggesting inflammation and possibly downstream effects on IP-10 play a greater role in microbiome mediated susceptibility to HIV.

## Author’s note

Membership of the KAVI-ICR team is provided in the Acknowledgments.

## Data availability statement

Microbiome sequencing reads and associated data have been deposited on NCBI BioProject under accession: PRJNA898823.The raw data supporting the conclusions of this manuscript will be made available by the authors, without undue reservation, to any qualified researcher.

## Ethics statement

The studies involving human participants were reviewed and approved by Kenyatta National Hospital/University of Nairobi Ethics and Research Committee, the University of Toronto Research Board and the Kenyan Pharmacy and Poisons Board. The patients/participants provided their written informed consent to participate in this study.

## Author contributions

KSM WJ, and CP conceived, designed, and coordinated the clinical and laboratory study. KAVI-ICR, MR, CP, JR, and ES contributed to methods, sample collection, processing and/or database curation. ES, SV, and JH performed or supervised the bioinformatic processing of the sequencing data. ES performed statistical analysis. ES, CP, RT, LRM, and KSM contributed to the analysis or interpretation of the results. ES wrote the initial version of this manuscript. ES, CP, JR, RT, SV, JH, LRM, PS, and KSM, were responsible for the critical revision of this manuscript. All authors contributed to the article and approved the submitted version.

## References

[1] Delgado-Diaz DJ Tyssen D Hayward JA Gugasyan R Hearps AC Tachedjian G. Distinct immune responses elicited from cervicovaginal epithelial cells by lactic acid and short chain fatty acids associated with optimal and non-optimal vaginal microbiota. Front Cell Infect Microbiol (2020) 9:446. doi: 10.3389/fcimb.2019.00446 31998660 PMC6965070

[2] Hearps AC Tyssen D Srbinovski D Bayigga L Diaz DJD Aldunate M. Vaginal lactic acid elicits an anti-inflammatory response from human cervicovaginal epithelial cells and inhibits production of pro-inflammatory mediators associated with HIV acquisition. Mucosal Immunol (2017) 10(6):1480–90. doi: 10.1038/mi.2017.27 28401934

[3] McKinnon LR Achilles SL Bradshaw CS Burgener A Crucitti T Fredricks DN. The evolving facets of bacterial vaginosis: Implications for HIV transmission. AIDS Res Hum Retroviruses (2019) 35(3):219–28. doi: 10.1089/aid.2018.0304 PMC6434601 30638028

[4] Petrova MI Reid G Vaneechoutte M Lebeer S. Lactobacillus iners: Friend or foe? Trends Microbiol (2017) 25(3):182–91. doi: 10.1016/j.tim.2016.11.007 27914761

[5] Vaneechoutte M. Lactobacillus iners, the unusual suspect. Res Microbiol (2017) 168(9-10):826–36. doi: 10.1016/j.resmic.2017.09.003 28951208

[6] Spiegel CA Amsel R Holmes KK. Diagnosis of bacterial vaginosis by direct gram stain of vaginal fluid. J Clin Microbiol (1983) 18(1):170–7. doi: 10.1128/jcm.18.1.170-177.1983 PMC270763 6193137

[7] Rajalakshmi R Kalaivani S. Prevalence of asymptomatic infections in sexually transmitted diseases attendees diagnosed with bacterial vaginosis, vaginal candidiasis, and trichomoniasis. Indian J Sex Transm Dis AIDS. (2016) 37(2):139–42. doi: 10.4103/0253-7184.192121 PMC5111297 27890946

[8] Bagnall P Rizzolo D. Bacterial vaginosis: A practical review. JAAPA (2017) 30(12):15–21. doi: 10.1097/01.JAA.0000526770.60197.fa 29135564

[9] Atashili J Poole C Ndumbe PM Adimora AA Smith JS. Bacterial vaginosis and HIV acquisition: a meta-analysis of published studies. AIDS (2008) 22(12):1493–501. doi: 10.1097/QAD.0b013e3283021a37 PMC2788489 18614873

[10] Allsworth JE Peipert JF. Severity of bacterial vaginosis and the risk of sexually transmitted infection. Am J Obstet Gynecol. (2011) 205(2):113.e1–.e6. doi: 10.1016/j.ajog.2011.02.060 PMC3156883 21514555

[11] Cohen CR Lingappa JR Baeten JM Ngayo MO Spiegel CA Hong T. Bacterial vaginosis associated with increased risk of female-to-Male HIV-1 transmission: A prospective cohort analysis among African couples. PloS Med (2012) 9(6):e1001251. doi: 10.1371/journal.pmed.1001251 22745608 PMC3383741

[12] Gosmann C Anahtar MN Handley SA Farcasanu M Abu-Ali G Bowman BA. Lactobacillus-deficient cervicovaginal bacterial communities are associated with increased HIV acquisition in young south African women. Immunity (2017) 46(1):29–37. doi: 10.1016/j.immuni.2016.12.013 28087240 PMC5270628

[13] McClelland RS Lingappa JR Srinivasan S Kinuthia J John-Stewart GC Jaoko W. Evaluation of the association between the concentrations of key vaginal bacteria and the increased risk of HIV acquisition in African women from five cohorts: a nested case-control study. Lancet Infect Dis (2018) 18(5):554–64. doi: 10.1016/S1473-3099(18)30058-6 PMC6445552 29396006

[14] Wasiela M Krzemiński Z Kalinka J Brzezińska-Błaszczyk E. [Correlation between levels of selected cytokines in cervico-vaginal fluid of women with abnormal vaginal bacterial flora]. Med Dosw Mikrobiol. (2005) 57(3):327–33. 16494210

[15] Thurman AR Kimble T Herold B Mesquita PM Fichorova RN Dawood HY. Bacterial vaginosis and subclinical markers of genital tract inflammation and mucosal immunity. AIDS Res Hum Retroviruses (2015) 31(11):1139–52. doi: 10.1089/aid.2015.0006 PMC4651020 26204200

[16] Masson L Arnold KB Little F Mlisana K Lewis DA Mkhize N. Inflammatory cytokine biomarkers to identify women with asymptomatic sexually transmitted infections and bacterial vaginosis who are at high risk of HIV infection. Sex Transm Infect (2016) 92(3):186–93. doi: 10.1136/sextrans-2015-052072 PMC6801014 26511781

[17] Jespers V Kyongo J Joseph S Hardy L Cools P Crucitti T. A longitudinal analysis of the vaginal microbiota and vaginal immune mediators in women from sub-Saharan Africa. Sci Rep (2017) 7(1):11974. doi: 10.1038/s41598-017-12198-6 28931859 PMC5607244

[18] Perciani CT Farah B Kaul R Ostrowski MA Mahmud SM Anzala O. Live attenuated varicella-zoster virus vaccine does not induce HIV target cell activation. J Clin Invest. (2019) 129(2):875–86. doi: 10.1172/JCI124473 PMC6355215 30511963

[19] Masson L Barnabas S Deese J Lennard K Dabee S Gamieldien H. Inflammatory cytokine biomarkers of asymptomatic sexually transmitted infections and vaginal dysbiosis: a multicentre validation study. Sex Transm Infect (2019) 95(1):5–12. doi: 10.1136/sextrans-2017-053506 30018088

[20] Masson L Passmore JA Liebenberg LJ Werner L Baxter C Arnold KB. Genital inflammation and the risk of HIV acquisition in women. Clin Infect Dis (2015) 61(2):260–9. doi: 10.1093/cid/civ298 PMC4565995 25900168

[21] McKinnon LR Liebenberg LJ Yende-Zuma N Archary D Ngcapu S Sivro A. Genital inflammation undermines the effectiveness of tenofovir gel in preventing HIV acquisition in women. Nat Med (2018) 24(4):491–6. doi: 10.1038/nm.4506 PMC5893390 29480895

[22] Anahtar MN Byrne EH Doherty KE Bowman BA Yamamoto HS Soumillon M. Cervicovaginal bacteria are a major modulator of host inflammatory responses in the female genital tract. Immunity (2015) 42(5):965–76. doi: 10.1016/j.immuni.2015.04.019 PMC4461369 25992865

[23] Dabee S Barnabas SL Lennard KS Jaumdally SZ Gamieldien H Balle C. Defining characteristics of genital health in south African adolescent girls and young women at high risk for HIV infection. PloS One (2019) 14(4):e0213975. doi: 10.1371/journal.pone.0213975 30947260 PMC6448899

[24] Lennard K Dabee S Barnabas SL Havyarimana E Blakney A Jaumdally SZ. Microbial composition predicts genital tract inflammation and persistent bacterial vaginosis in south African adolescent females. Infect Immun (2017) 86(1):e00410–17. doi: 10.1128/IAI.00410-17 PMC5736802 29038128

[25] Bradshaw CS Sobel JD. Current treatment of bacterial vaginosis-limitations and need for innovation. J Infect Dis (2016) 214 Suppl 1(Suppl 1):S14–20. doi: 10.1093/infdis/jiw159 PMC4957510 27449869

[26] Bradshaw CS Pirotta M De Guingand D Hocking JS Morton AN Garland SM. Efficacy of oral metronidazole with vaginal clindamycin or vaginal probiotic for bacterial vaginosis: randomised placebo-controlled double-blind trial. PloS One (2012) 7(4):e34540. doi: 10.1371/journal.pone.0034540 22509319 PMC3317998

[27] Bradshaw CS Morton AN Hocking J Garland SM Morris MB Moss LM. High recurrence rates of bacterial vaginosis over the course of 12 months after oral metronidazole therapy and factors associated with recurrence. J Infect Dis (2006) 193(11):1478–86. doi: 10.1086/503780 16652274

[28] Mtshali A San JE Osman F Garrett N Balle C Giandhari J. Temporal changes in vaginal microbiota and genital tract cytokines among south African women treated for bacterial vaginosis. Front Immunol (2021) 12:730986. doi: 10.3389/fimmu.2021.730986 34594336 PMC8477043

[29] Hickey RJ Forney LJ. Gardnerella vaginalis does not always cause bacterial vaginosis. J Infect Dis (2014) 210(10):1682–3. doi: 10.1093/infdis/jiu303 PMC4334793 24855684

[30] Schwebke JR Muzny CA Josey WE. Role of gardnerella vaginalis in the pathogenesis of bacterial vaginosis: a conceptual model. J Infect Dis (2014) 210(3):338–43. doi: 10.1093/infdis/jiu089 24511102

[31] Gilbert NM Lewis WG Li G Sojka DK Lubin JB Lewis AL. Gardnerella vaginalis and prevotella bivia trigger distinct and overlapping phenotypes in a mouse model of bacterial vaginosis. J Infect Dis (2019) 220(7):1099–108. doi: 10.1093/infdis/jiy704 PMC6736442 30715405

[32] Muzny CA Taylor CM Swords WE Tamhane A Chattopadhyay D Cerca N. An updated conceptual model on the pathogenesis of bacterial vaginosis. J Infect Dis (2019) 220(9):1399–405. doi: 10.1093/infdis/jiz342 PMC6761952 31369673

[33] Morrill S Gilbert NM Lewis AL. Gardnerella vaginalis as a cause *of bacterial vaginosi*s: Appraisal of the evidence from *in vivo* models. Front Cell Infect Microbiol (2020) 10:168. doi: 10.3389/fcimb.2020.00168 32391287 PMC7193744

[34] Ahmed A Earl J Retchless A Hillier SL Rabe LK Cherpes TL. Comparative genomic analyses of 17 clinical isolates of gardnerella vaginalis provide evidence of multiple genetically isolated clades consistent with subspeciation into genovars. J Bacteriol (2012) 194(15):3922–37. doi: 10.1128/JB.00056-12 PMC3416530 22609915

[35] Vaneechoutte M Guschin A Van Simaey L Gansemans Y Van Nieuwerburgh F Cools P. Emended description of gardnerella vaginalis and description of gardnerella leopoldii sp. nov., gardnerella piotii sp. nov. and gardnerella swidsinskii sp. nov., with delineation of 13 genomic species within the genus gardnerella. Int J Syst Evol Microbiol (2019) 69(3):679–87. doi: 10.1099/ijsem.0.003200 30648938

[36] Hill JE Albert AYK Group VR. Resolution and cooccurrence patterns of gardnerella leopoldii, G. swidsinskii G. piotii G. vaginalis within Vaginal Microbiome. Infect Immun (2019) 87(12):e00532–19. doi: 10.1128/IAI.00532-19 PMC6867840 31527125

[37] Paramel Jayaprakash T Schellenberg JJ Hill JE. Resolution and characterization of distinct cpn60-based subgroups of gardnerella vaginalis in the vaginal microbiota. PloS One (2012) 7(8):e43009. doi: 10.1371/journal.pone.0043009 22900080 PMC3416817

[38] Schellenberg JJ Paramel Jayaprakash T Withana Gamage N Patterson MH Vaneechoutte M Hill JE. Gardnerella vaginalis subgroups defined by cpn60 sequencing and sialidase activity in isolates from Canada, Belgium and Kenya. PloS One (2016) 11(1):e0146510. doi: 10.1371/journal.pone.0146510 26751374 PMC4709144

[39] Cornejo OE Hickey RJ Suzuki H Forney LJ. Focusing the diversity of gardnerella vaginalis through the lens of ecotypes. Evol Appl (2017) 11(3):312–24. doi: 10.1111/eva.12555 PMC5881158 29632552

[40] Janulaitiene M Gegzna V Baranauskiene L Bulavaite A Simanavicius M Pleckaityte M. Phenotypic characterization of gardnerella vaginalis subgroups suggests differences in their virulence potential. PloS One (2018) 13(7):e0200625. doi: 10.1371/journal.pone.0200625 30001418 PMC6042761

[41] Khan S Voordouw MJ Hill JE. Competition among gardnerella subgroups from the human vaginal microbiome. Front Cell Infect Microbiol (2019) 9:374. doi: 10.3389/fcimb.2019.00374 31737577 PMC6834547

[42] Khan S Vancuren SJ Hill JE. A generalist lifestyle allows rare gardnerella spp. to persist at low levels in the vaginal microbiome. Microb Ecol (2021) 82(4):1048–60. doi: 10.1007/s00248-020-01643-1 PMC7678777 33219399

[43] Eade CR Diaz C Wood MP Anastos K Patterson BK Gupta P. Identification and characterization of bacterial vaginosis-associated pathogens using a comprehensive cervical-vaginal epithelial coculture assay. PloS One (2012) 7(11):e50106. doi: 10.1371/journal.pone.0050106 23166828 PMC3499514

[44] Garcia EM Kraskauskiene V Koblinski JE Jefferson KK. Interaction of gardnerella vaginalis and vaginolysin with the apical versus basolateral face of a three-dimensional model of vaginal epithelium. Infect Immun (2019) 87(4):e00646–18. doi: 10.1128/IAI.00646-18 PMC6434120 30692180

[45] Perciani CT Jaoko W Walmsley S Farah B Mahmud SM Ostrowski M. Protocol of a randomised controlled trial characterising the immune responses induced by varicella-zoster virus (VZV) vaccination in healthy Kenyan women: setting the stage for a potential VZV-based HIV vaccine. BMJ Open (2017) 7(9):e017391. doi: 10.1136/bmjopen-2017-017391 PMC5623463 28939581

[46] Nugent RP Krohn MA Hillier SL. Reliability of diagnosing bacterial vaginosis is improved by a standardized method of gram stain interpretation. J Clin Microbiol (1991) 29(2):297–301. doi: 10.1128/jcm.29.2.297-301.1991 1706728 PMC269757

[47] Shvartsman E Richmond MEI Schellenberg JJ Lamont A Perciani C Russell JNH. Comparative analysis of DNA extraction and PCR product purification methods for cervicovaginal microbiome analysis using cpn60 microbial profiling. PloS One (2022) 17(1):e0262355. doi: 10.1371/journal.pone.0262355 35025956 PMC8758110

[48] Fernando C Hill JE. cpn60 metagenomic amplicon library preparation for the illumina miseq platform. Protocol Exchange (2021). doi: 10.21203/rs.3.pex-1438/v1

[49] Vancuren SJ Dos Santos SJ Hill JE Team MMLP. Evaluation of variant calling for cpn60 barcode sequence-based microbiome profiling. PloS One (2020) 15(7):e0235682. doi: 10.1371/journal.pone.0235682 32645030 PMC7347135

[50] Callahan BJ McMurdie PJ Rosen MJ Han AW Johnson AJ Holmes SP. DADA2: High-resolution sample inference from illumina amplicon data. Nat Methods (2016) 13(7):581–3. doi: 10.1038/nmeth.3869 PMC4927377 27214047

[51] Johnson LA Chaban B Harding JC Hill JE. Optimizing a PCR protocol for cpn60-based microbiome profiling of samples variously contaminated with host genomic DNA. BMC Res Notes. (2015) 8:253. doi: 10.1186/s13104-015-1170-4 26092180 PMC4475309

[52] Perciani CT Jaoko W Farah B Ostrowski MA Anzala O MacDonald KS. αEβ7, α4β7 and α4β1 integrin contributions to T cell distribution in blood, cervix and rectal tissues: Potential implications for HIV transmission. PloS One (2018) 13(2):e0192482. doi: 10.1371/journal.pone.0192482 29420608 PMC5805330

[53] Luke SG. Evaluating significance in linear mixed-effects models in r. Behav Res Methods (2017) 49(4):1494–502. doi: 10.3758/s13428-016-0809-y 27620283

[54] Bayigga L Nabatanzi R Ssekagiri A Kateete DP Sekikubo M Anderson DJ. Diverse vaginal microbiome was associated with pro-inflammatory vaginal milieu among pregnant women in Uganda. Hum Microbiome J (2020) 18:100076. doi: 10.1016/j.humic.2020.100076

[55] Sivro A Mwatelah R Kambaran C Gebrebrhan H Becker MG Ma H. Sex work is associated with increased vaginal microbiome diversity in young women from Mombasa, Kenya. J Acquir Immune Defic Syndr (2020) 85(1):79–87. doi: 10.1097/QAI.0000000000002406 32433252 PMC12506801

[56] Shipitsyna E Krysanova A Khayrullina G Shalepo K Savicheva A Guschin A. Quantitation of all four gardnerella vaginalis clades detects abnormal vaginal microbiota characteristic of bacterial vaginosis more accurately than putative g. vaginalis sialidase a gene count. Mol Diagn Ther (2019) 23(1):139–47. doi: 10.1007/s40291-019-00382-5 PMC6394432 30721449

[57] Janulaitiene M Paliulyte V Grinceviciene S Zakareviciene J Vladisauskiene A Marcinkute A. Prevalence and distribution of gardnerella vaginalis subgroups in women with and without bacterial vaginosis. BMC Infect Dis (2017) 17(1):394. doi: 10.1186/s12879-017-2501-y 28583109 PMC5460423

[58] Cole AM Ganz T Liese AM Burdick MD Liu L Strieter RM. Cutting edge: IFN-inducible ELR- CXC chemokines display defensin-like antimicrobial activity. J Immunol (2001) 167(2):623–7. doi: 10.4049/jimmunol.167.2.623 11441062

[59] Yang D Chen Q Hoover DM Staley P Tucker KD Lubkowski J. Many chemokines including CCL20/MIP-3alpha display antimicrobial activity. J Leukoc Biol (2003) 74(3):448–55. doi: 10.1189/jlb.0103024 12949249

[60] Groom JR Luster AD. CXCR3 ligands: redundant, collaborative and antagonistic functions. Immunol Cell Biol (2011) 89(2):207–15. doi: 10.1038/icb.2010.158 PMC3863330 21221121

[61] Liu M Guo S Hibbert JM Jain V Singh N Wilson NO. CXCL10/IP-10 in infectious diseases pathogenesis and potential therapeutic implications. Cytokine Growth Factor Rev (2011) 22(3):121–30. doi: 10.1016/j.cytogfr.2011.06.001 PMC3203691 21802343

[62] Poggi A Zancolli M Catellani S Borsellino G Battistini L Zocchi MR. Migratory pathways of gammadelta T cells and response to CXCR3 and CXCR4 ligands: adhesion molecules involved and implications for multiple sclerosis pathogenesis. Ann N Y Acad Sci (2007) 1107:68–78. doi: 10.1196/annals.1381.008 17804534

[63] Alcaide ML Strbo N Romero L Jones DL Rodriguez VJ Arheart K. Bacterial vaginosis is associated with loss of gamma delta T cells in the female reproductive tract in women in the Miami women interagency HIV study (WIHS): A cross sectional study. PloS One (2016) 11(4):e0153045. doi: 10.1371/journal.pone.0153045 27078021 PMC4831836

[64] Barel O Aizenbud Y Tabib Y Jaber Y Leibovich A Horev Y. γδ T cells differentially regulate bone loss in periodontitis models. J Dent Res (2022) 101(4):428–36. doi: 10.1177/00220345211042830 34715745

[65] Joag V Obila O Gajer P Scott MC Dizzell S Humphrys M. Impact of standard bacterial vaginosis treatment on the genital microbiota, immune milieu, and ex vivo human immunodeficiency virus susceptibility. Clin Infect Dis (2019) 68(10):1675–83. doi: 10.1093/cid/ciy762 PMC6495022 30407498

