# Supplementary material for: Gardnerella subgroup dominant microbiomes are associated with divergent cervicovaginal immune responses in a longitudinal cohort of Kenyan women
Source: Front Immunol. 2023 Jan 16;13:974195. doi: 10.3389/fimmu.2022.974195 (PMC9886495; doi:10.3389/fimmu.2022.974195)

Appendix 1. Sample collection Schedule for KAVI-VZV-001 participants. Half of the participants also contributed additional samples during the visits shown in red. * indicates no immune activation data was processed from cervical cytobrush samples during that visit.

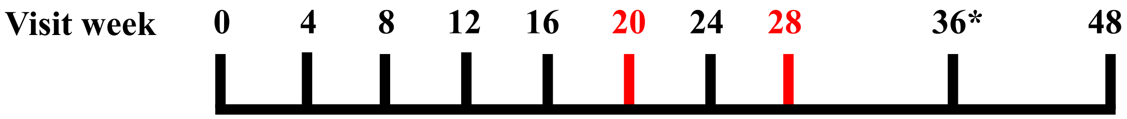

Appendix 2. Longitudinal demographics of KAVI-VZV-001 study participants

Table S1. Longitudinal demographics for the KAVI-VZV-001 trial participants included in this ancillary study.

Visit Week	0	4	8	12	16	20	24	28	36	48	Total Samples
**Participants**	40	40	41	41	41	20	41	20	40	38	362
**BV Status**											
Negative (Nugent 0-3), no (%)	30 (75)	32 (80)	30 (73.2)	34 (82.9)	34 (82.9)	15 (75)	31 (75.6)	16 (80)	31 (77.5)	28 (73.7)	281 (77.6)
Intermediate (Nugent 4-6), no (%)	7 (17.5)	4 (10)	7 (17.1)	5 (12.2)	4 (9.8)	3 (15)	6 (14.6)	3 (15)	4 (10)	4 (10.5)	47 (13)
Positive (Nugent 7-10), no (%)	3 (7.5)	4 (10)	4 (9.8)	2 (4.9)	3 (7.3)	2 (10)	4 (9.8)	1 (5)	5 (12.5)	6 (15.8)	34 (9.4)
**Contraceptive**											
DMPA, no (%)	24 (60)	25 (62.5)	24 (58.5)	24 (58.5)	24 (58.5)	11 (55)	24 (58.5)	11 (55)	23 (57.5)	23 (60.5)	213 (58.8)
Levonorgestrel, no (%)	8 (20)	8 (20)	8 (19.5)	8 (19.5)	8 (19.5)	3 (15)	8 (19.5)	3 (15)	8 (20)	7 (18.4)	69 (19.1)
Etonogestrel, no (%)	7 (17.5)	6 (15)	7 (17.1)	7 (17.1)	7 (17.1)	4 (20)	6 (14.6)	3 (15)	6 (15)	6 (15.8)	59 (16.3)
COC, no (%)	0 (0)	0 (0)	0 (0)	0 (0)	0 (0)	0 (0)	1 (2.4)	1 (5)	1 (2.5)	0 (0)	3 (0.8)
IUD, no (%)	1 (2.5)	1 (2.5)	2 (4.9)	2 (4.9)	2 (4.9)	2 (10)	2 (4.9)	2 (10)	2 (5)	2 (5.3)	18 (5)
**BV-Antibiotics**[a,*]											
Yes, no (%)	8 (20)	2 (5)	4 (9.8)	2 (4.9)	3 (7.3)	1 (5)	3 (7.3)	2 (10)	5 (12.5)	5 (13.2)	35 (9.7)
No, no (%)	32 (80)	38 (95)	37 (90.2)	39 (95.1)	38 (92.7)	19 (95)	38 (92.7)	18 (90)	35 (87.5)	33 (86.8)	327 (90.3)
**Other Anibiotics**[b,*]											
Yes, no (%)	8 (20)	4 (10)	6 (14.6)	7 (17.1)	5 (12.2)	2 (10)	8 (19.5)	5 (25)	9 (22.5)	13 (34.2)	67 (18.5)
No, no (%)	32 (80)	36 (90)	35 (85.4)	34 (82.9)	36 (87.8)	18 (90)	33 (80.5)	15 (75)	31 (77.5)	25 (65.8)	295 (81.5)
**Anti-fungals***											
Yes, no (%)	4 (10)	1 (2.5)	4 (9.8)	6 (14.6)	3 (7.3)	1 (5)	2 (4.9)	1 (5)	8 (20)	8 (21.1)	38 (10.5)
No, no (%)	36 (90)	39 (97.5)	37 (90.2)	35 (85.4)	38 (92.7)	19 (95)	39 (95.1)	19 (95)	32 (80)	30 (78.9)	324 (89.5)
**Vulvovaginal Candidasis**											
Positive, no (%)	3 (7.5)	3 (7.5)	4 (9.8)	1 (2.4)	2 (4.9)	1 (5)	4 (9.8)	1 (5)	3 (7.5)	1 (2.6)	23 (6.4)
Negative, no (%)	37 (92.5)	37 (92.5)	37 (90.2)	40 (97.6)	39 (95.1)	19 (95)	37 (90.2)	19 (95)	37 (92.5)	37 (97.4)	339 (93.6)
**HSV-2 status at enrollment**											
Positive, no (%)	23 (57.5)	23 (57.5)	24 (58.5)	24 (58.5)	24 (58.5)	16 (80)	24 (58.5)	16 (80)	23 (57.5)	22 (57.9)	219 (60.5)
Negative, no (%)	16 (40)	16 (40)	16 (39)	16 (39)	16 (39)	3 (15)	16 (39)	3 (15)	16 (40)	15 (39.5)	133 (36.7)
Indeterminate, no (%)	1 (2.5)	1 (2.5)	1 (2.4)	1 (2.4)	1 (2.4)	1 (5)	1 (2.4)	1 (5)	1 (2.5)	1 (2.6)	10 (2.8)
**CST**											
**Lactobacillus dominant**											
LC, no (%)	9 (22.5)	10 (25)	7 (17.1)	9 (22)	9 (22)	6 (30)	8 (19.5)	5 (25)	8 (20)	11 (28.9)	82 (22.7)
LJ, no (%)	2 (5)	6 (15)	5 (12.2)	5 (12.2)	4 (9.8)	1 (5)	2 (4.9)	3 (15)	7 (17.5)	4 (10.5)	39 (10.8)
LG, no (%)	1 (2.5)	1 (2.5)	1 (2.4)	1 (2.4)	1 (2.4)	0 (0)	1 (2.4)	0 (0)	1 (2.5)	0 (0)	7 (1.9)
LCo, no (%)	1 (2.5)	0 (0)	2 (4.9)	2 (4.9)	1 (2.4)	3 (15)	2 (4.9)	0 (0)	0 (0)	0 (0)	11 (3)
LO, no (%)	2 (5)	0 (0)	0 (0)	2 (4.9)	1 (2.4)	1 (5)	1 (2.4)	0 (0)	1 (2.5)	1 (2.6)	9 (2.5)
LI, no (%)	11 (27.5)	11 (27.5)	11 (26.8)	9 (22)	13 (31.7)	3 (15)	9 (22)	5 (25)	11 (27.5)	10 (26.3)	93 (25.7)
**Non-Lactobacillus dominance**											
GVA, no (%)	4 (10)	4 (10)	8 (19.5)	4 (9.8)	6 (14.6)	2 (10)	8 (19.5)	3 (15)	3 (7.5)	4 (10.5)	46 (12.7)
GVB, no (%)	2 (5)	3 (7.5)	4 (9.8)	3 (7.3)	1 (2.4)	0 (0)	0 (0)	0 (0)	0 (0)	1 (2.6)	14 (3.9)
GVC, no (%)	1 (2.5)	1 (2.5)	2 (4.9)	1 (2.4)	3 (7.3)	1 (5)	5 (12.2)	3 (15)	3 (7.5)	1 (2.6)	21 (5.8)
MIXED, no (%)	7 (17.5)	4 (10)	1 (2.4)	5 (12.2)	2 (4.9)	3 (15)	5 (12.2)	1 (5)	6 (15)	6 (15.8)	40 (11)

Abbreviations: DMPA-depot medroxyprogesterone acetate, COC-combined oral contraceptives, IUD-copper intra-uterine device, CST-community state type, LC - *L. crispatus* dominance, LJ - *L. jensenii* dominance, LG - *L. gasseri*, LCo - *L. coleohominis*, LO - *Latobacillus* other species, LI - *L. iners*, GVA - *Gardnarella* subgroup A dominance, GVB - *Gardnerella* subgroup B dominance, GVC - *Gardnerella* subgroup C dominance, MIXED-polymicrobial communities. [a]BV-antibiotics include tinidazole, clindamycin, metronidazole, or secnidazole. [b]does not include ophthalmic or external topical antibiotics. *Antimicrobials given in the period prior to the visit sample collection.

Appendix 3. Cervicovaginal microbial communities exhibit variable associations with log10-normalized cytokines. Linear mixed model estimates for microbial structure associations with (A) IFN-γ, (B) TNF-α, (C) IL-17, (D) IL-6, (E) IL-4, (F) IL-10, and (G) GM-CSF. The β-coefficients represent the mean change in specific log10-transformed cytokine concentration that is associated with a specific microbiome type (CST_agg) compared to Lactobacillus (non-iners) dominant microbial communities (LDo). Blue symbols represent unadjusted models only utilizing microbial groupings as predictors. Red symbols represent models following multivariable adjustment for hormonal contraceptive type, HSV-II seropositivity at baseline, concomitant vulvovaginal candidiasis, age, use of BV antibiotics, use of other antibiotics, and use of antifungals prior to sample collection. The error bars represent the 95% confidence intervals associated with the estimates from the linear mixed models. *P≤0.05, **P≤0.01, ***P≤0.001, ****P≤0.0001.

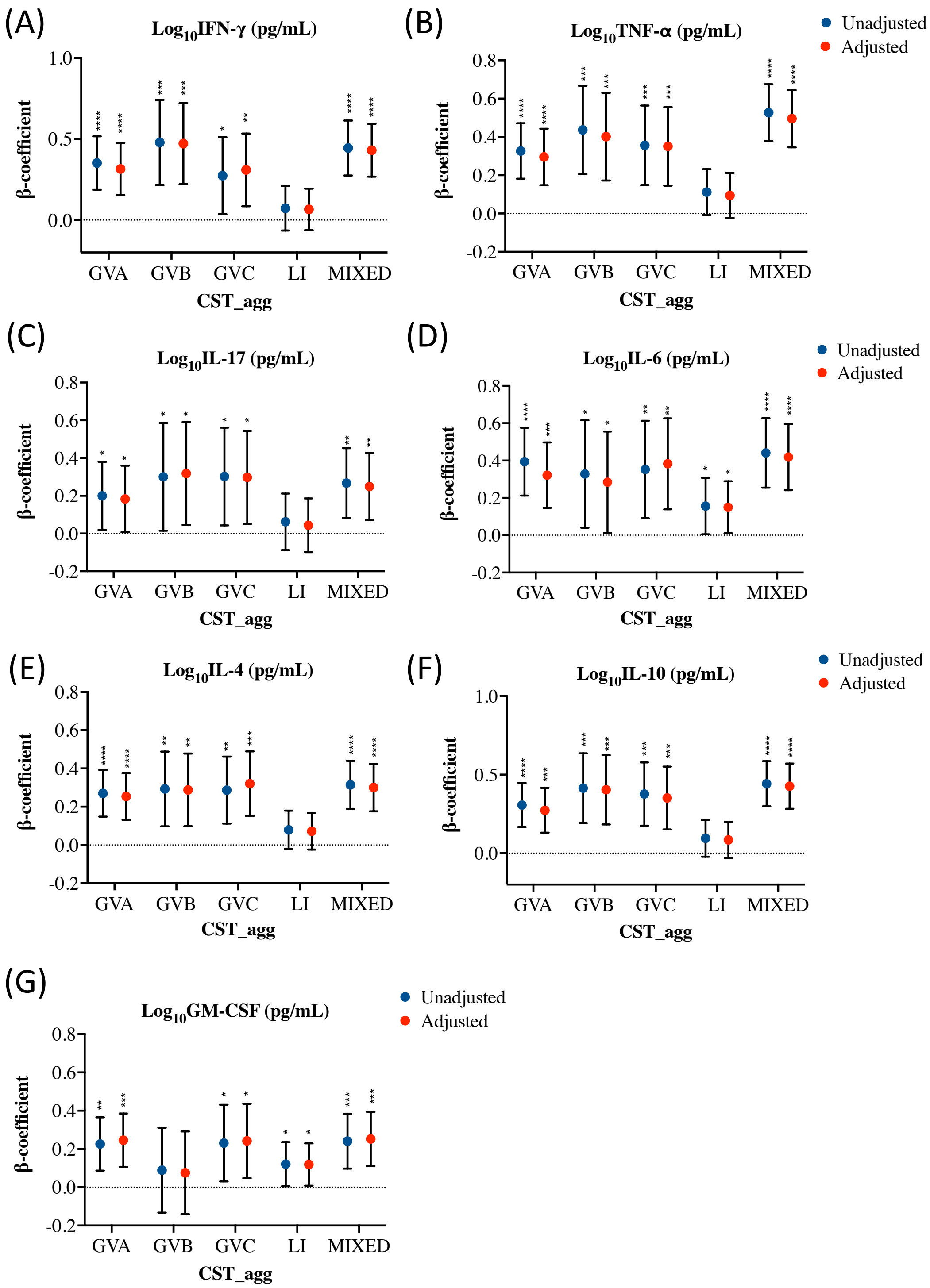

Appendix 4. Cervicovaginal microbial communities exhibit variable associations with log10-normalized chemokine concentrations. Linear mixed model estimates for microbial structure associations with (A) MIP-1α, (B) MIP-1β, (C) IL-8, and (D) MIP-3α. The β-coefficients represent the mean change in specific log10-transformed chemokine concentration that is associated with a specific microbiome type (CST_agg) compared to Lactobacillus (non-iners) dominant microbial communities (LDo). Blue symbols represent unadjusted models only utilizing microbial groupings as predictors. Red symbols represent models following multivariable adjustment for hormonal contraceptive type, HSV-II seropositivity at baseline, concomitant vulvovaginal candidiasis, age, use of BV antibiotics, use of other antibiotics, and use of antifungals prior to sample collection. The error bars represent the 95% confidence intervals associated with the estimates from the linear mixed models. *P≤0.05, **P≤0.01, ***P≤0.001, ****P≤0.0001.

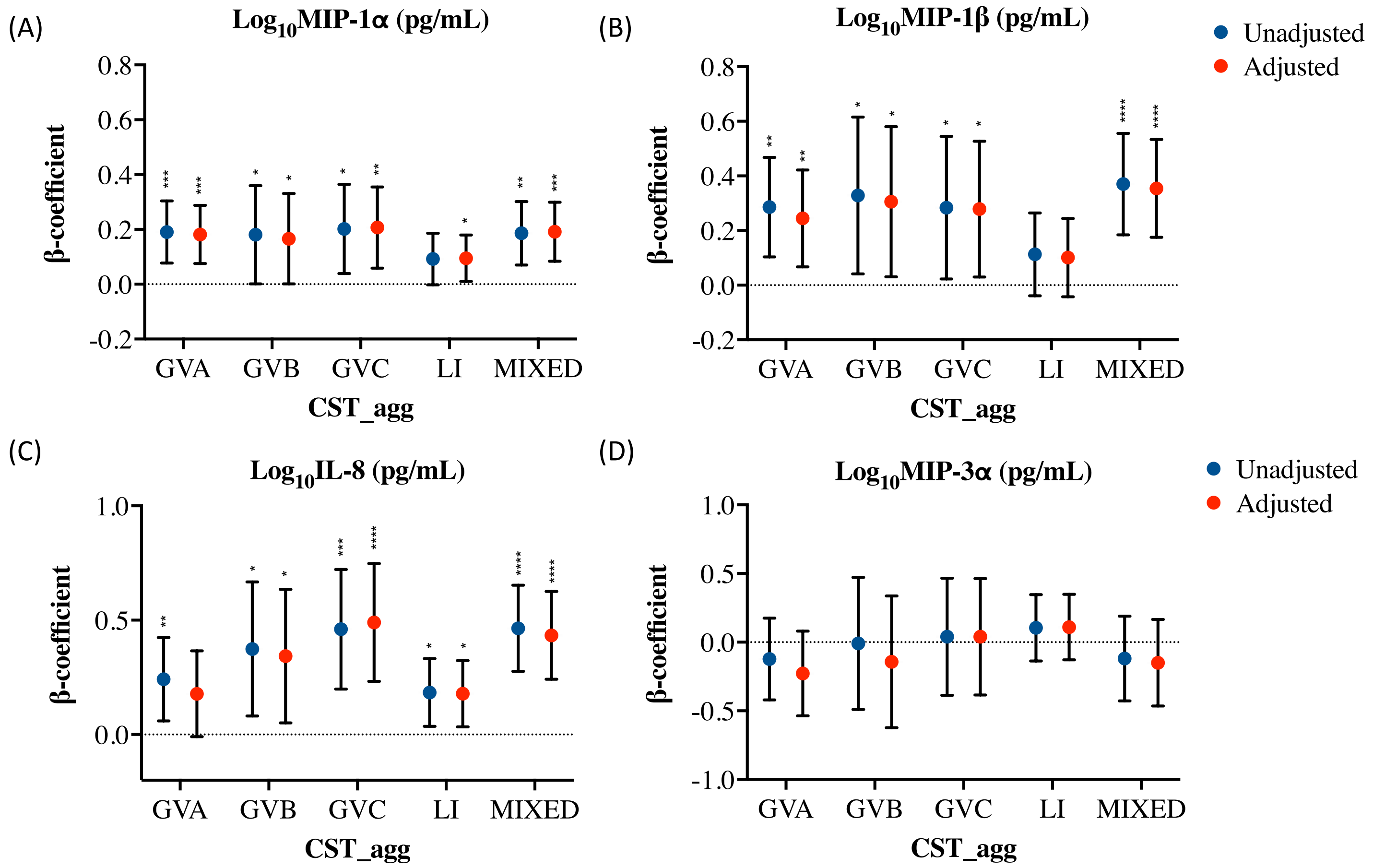

Appendix 5. Longitudinal changes in microbial community structures, and the three cytokine principal component scores in 39 study participants. CST_agg refers to aggregated community state types. Only the 25 most prevalent microbial taxa are shown with the rest of the species grouped as "other_species" and "Lactobacillus_other_species".

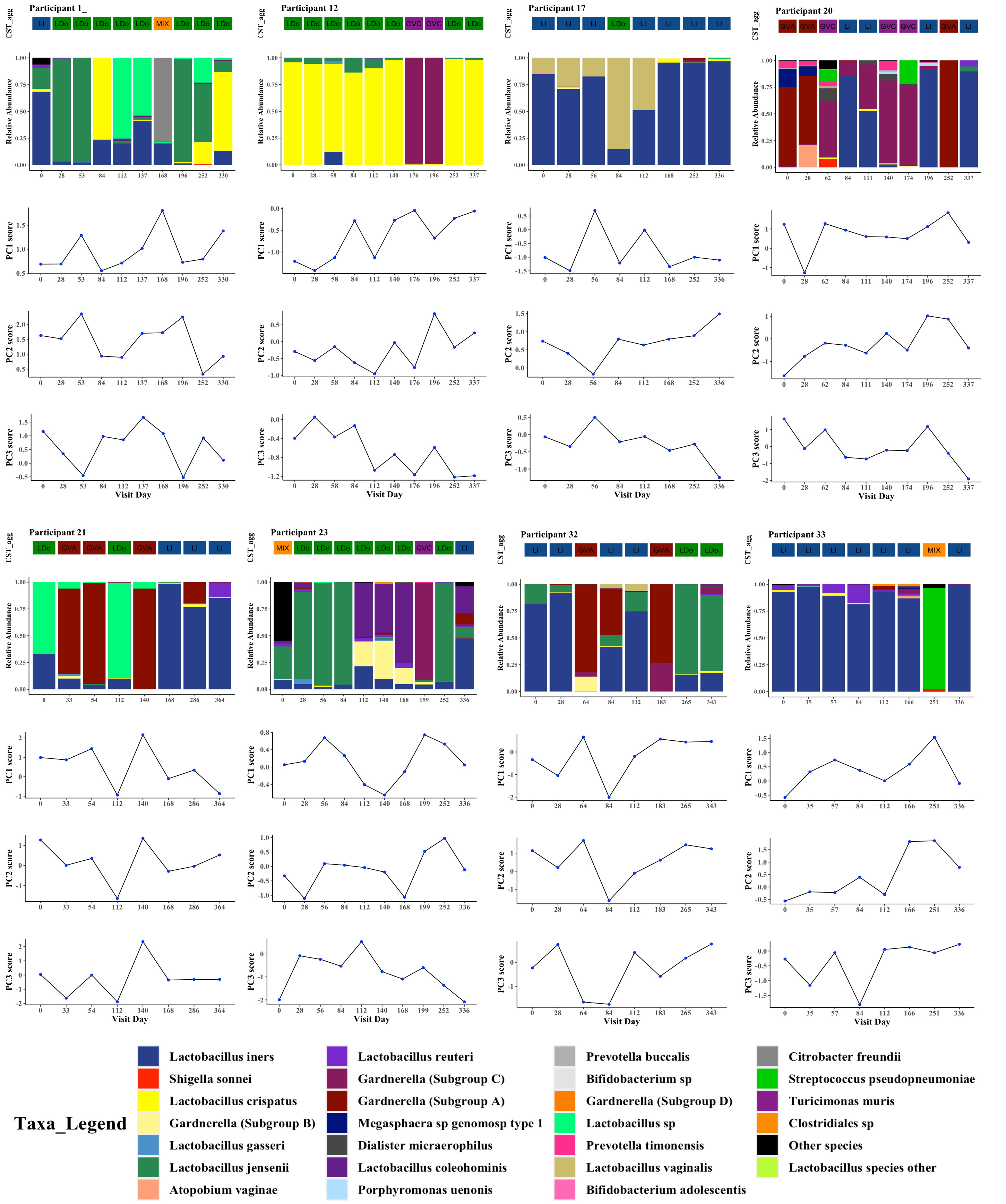

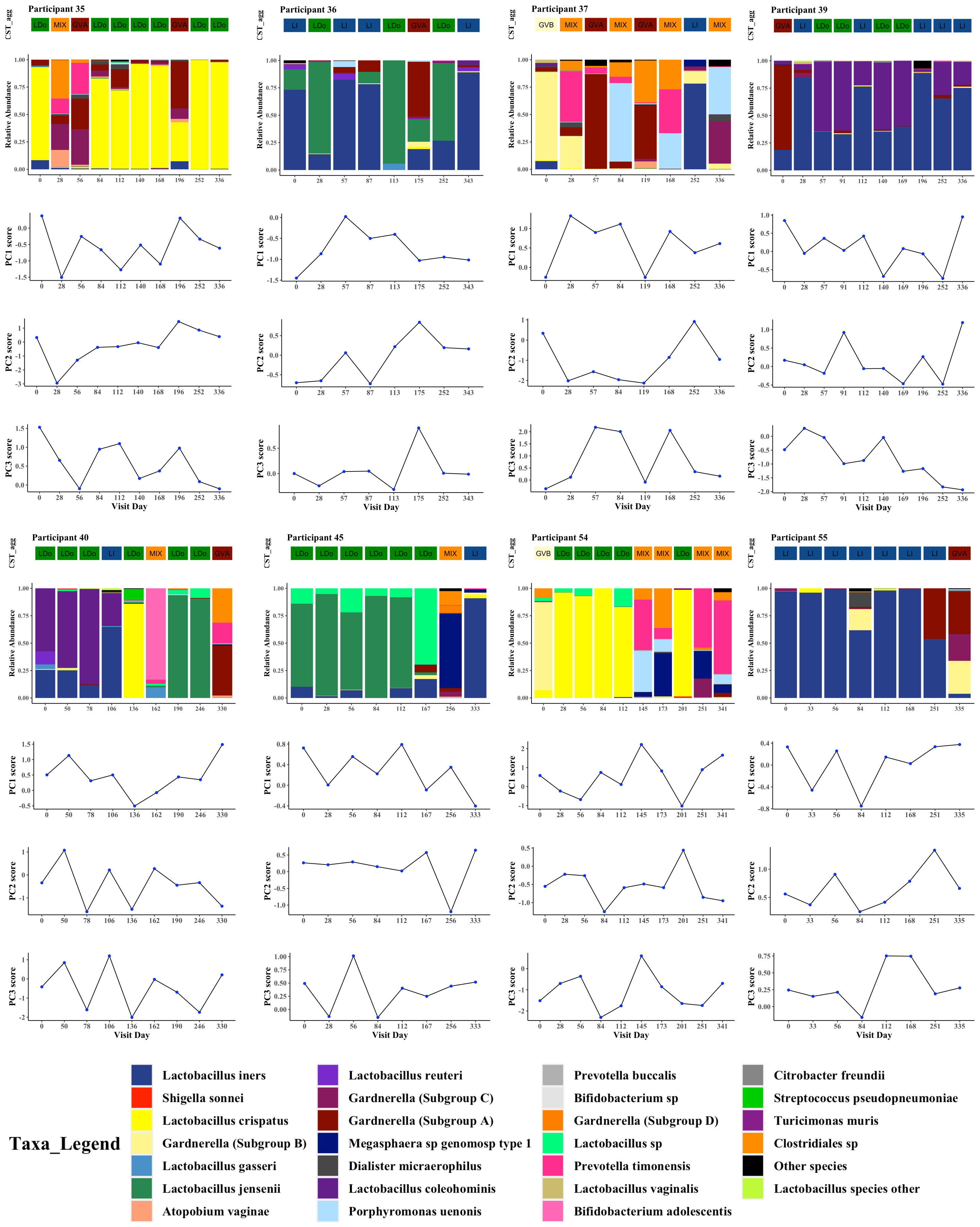
Participant 35
Participant 36
Participant 37
Participant 39
Participant 40
Participant 45
Participant 54
Participant 55
CST_agg
Relative Abundance
PC1 score
PC2 score
PC3 score
Visit Day
Taxa_Legend
Lactobacillus iners
Shigella sonnei
Lactobacillus crispatus
Gardnerella (Subgroup B)
Lactobacillus gasseri
Lactobacillus jensenii
Atopobium vaginae
Lactobacillus reuteri
Gardnerella (Subgroup C)
Gardnerella (Subgroup A)
Megasphaera sp genomosp type 1
Dialister micraerophilus
Lactobacillus coleohominis
Porphyromonas uenonis
Prevotella buccalis
Bifidobacterium sp
Gardnerella (Subgroup D)
Lactobacillus sp
Prevotella timonensis
Lactobacillus vaginalis
Bifidobacterium adolescentis
Citrobacter freundii
Streptococcus pseudopneumoniae
Turicimonas muris
Clostridiales sp
Other species
Lactobacillus species other

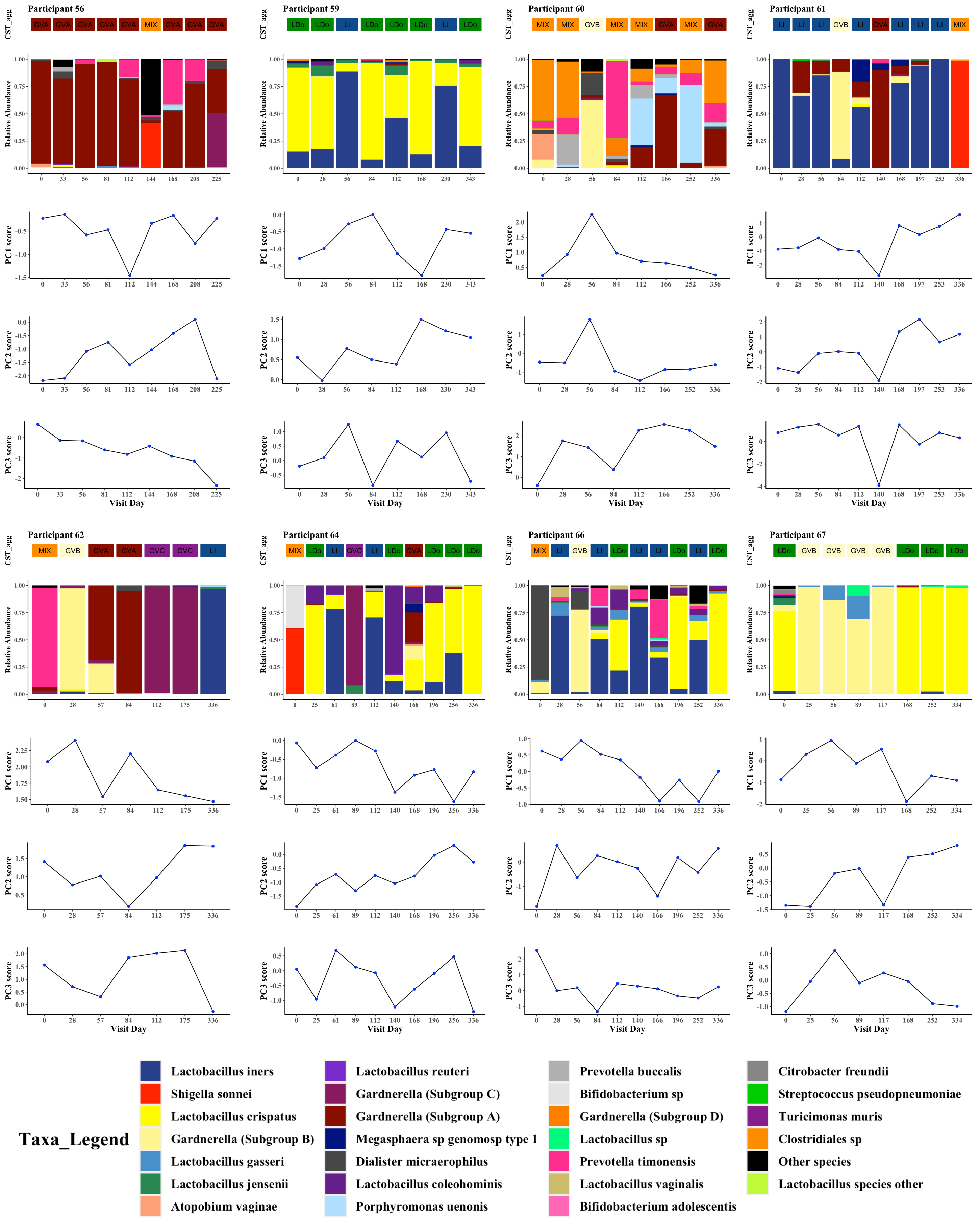

Participant 56
CST_agg
GVA GVA GVA GVA GVA MIX GVA GVA GVA
Relative Abundance
PC1 score
PC2 score
PC3 score
Visit Day
Participant 59
LDo LDo LI LDo LDo LDo LI LDo
Participant 60
MIX MIX GVB MIX MIX GVA MIX GVA
Participant 61
LI LI LI GVB LI GVA LI LI LI MIX
Participant 62
MIX GVB GVA GVA GVC GVC LI
Participant 64
MIX LDo LI GVC LI LDo GVA LDo LDo LDo
Participant 66
MIX LI GVB LI LDo LI LI LDo LI LDo
Participant 67
LDo GVB GVB GVB GVB LDo LDo LDo
Taxa_Legend
Lactobacillus iners
Shigella sonnei
Lactobacillus crispatus
Gardnerella (Subgroup B)
Lactobacillus gasseri
Lactobacillus jensenii
Atopobium vaginae
Lactobacillus reuteri
Gardnerella (Subgroup C)
Gardnerella (Subgroup A)
Megasphaera sp genomosp type 1
Dialister micraerophilus
Lactobacillus coleohominis
Porphyromonas uenonis
Prevotella buccalis
Bifidobacterium sp
Gardnerella (Subgroup D)
Lactobacillus sp
Prevotella timonensis
Lactobacillus vaginalis
Bifidobacterium adolescentis
Citrobacter freundii
Streptococcus pseudopneumoniae
Turicimonas muris
Clostridiales sp
Other species
Lactobacillus species other

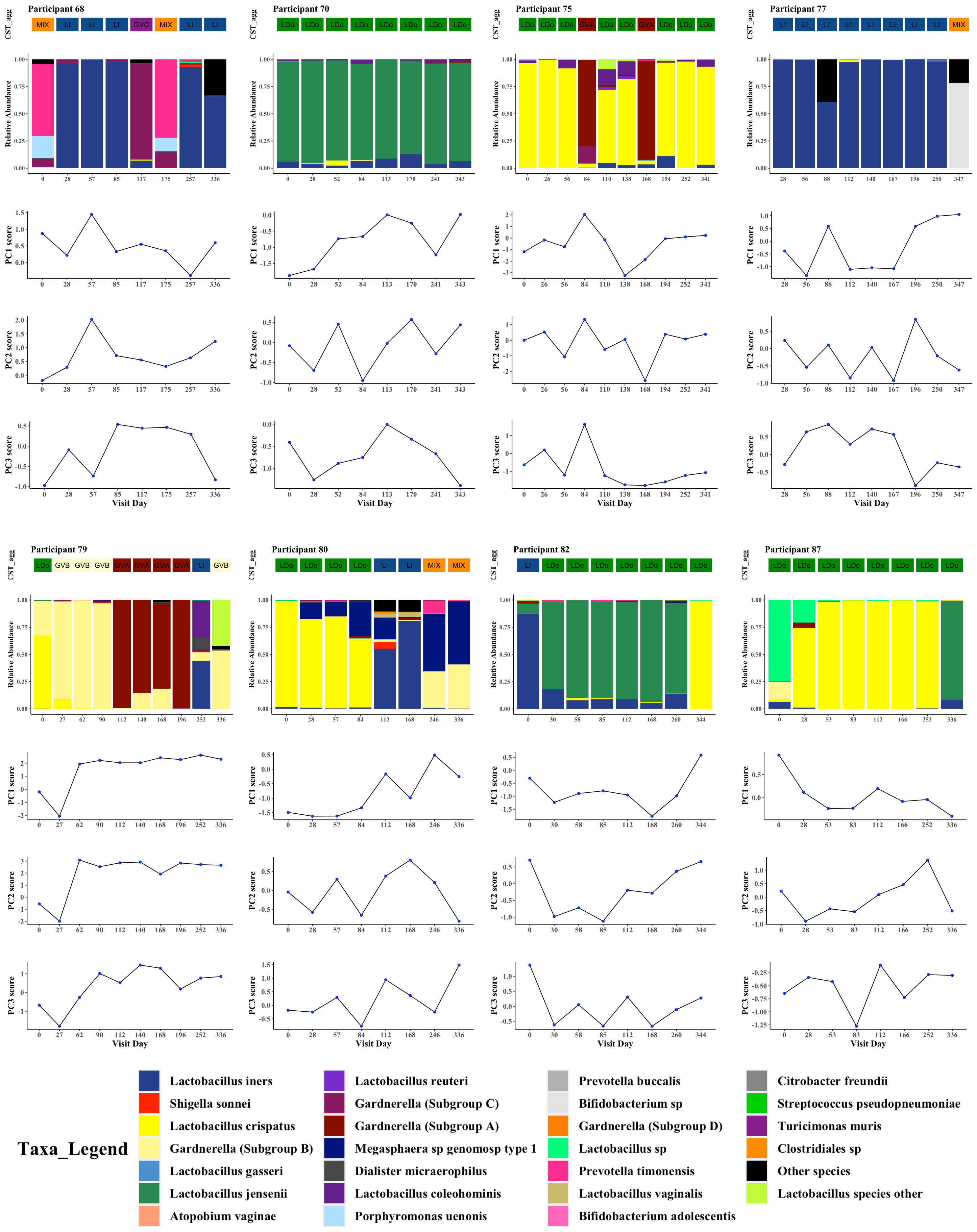

Participant 68
MIX LI LI LI GVC MIX LI LI
Participant 70
LDo LDo LDo LDo LDo LDo LDo LDo
Participant 75
LDo LDo LDo GVA LDo LDo GVA LDo LDo LDo
Participant 77
LI LI LI LI LI LI LI LI MIX
Participant 79
LDo GVB GVB GVB GVA GVA GVA GVA LI GVB
Participant 80
LDo LDo LDo LDo LI LI MIX MIX
Participant 82
LI LDo LDo LDo LDo LDo LDo LDo
Participant 87
LDo LDo LDo LDo LDo LDo LDo LDo
CST_agg
Relative Abundance
PC1 score
PC2 score
PC3 score
Visit Day
Taxa_Legend
Lactobacillus iners
Shigella sonnei
Lactobacillus crispatus
Gardnerella (Subgroup B)
Lactobacillus gasseri
Lactobacillus jensenii
Atopobium vaginae
Lactobacillus reuteri
Gardnerella (Subgroup C)
Gardnerella (Subgroup A)
Megasphaera sp genomosp type 1
Dialister micraerophilus
Lactobacillus coleohominis
Porphyromonas uenonis
Prevotella buccalis
Bifidobacterium sp
Gardnerella (Subgroup D)
Lactobacillus sp
Prevotella timonensis
Lactobacillus vaginalis
Bifidobacterium adolescentis
Citrobacter freundii
Streptococcus pseudopneumoniae
Turicimonas muris
Clostridiales sp
Other species
Lactobacillus species other

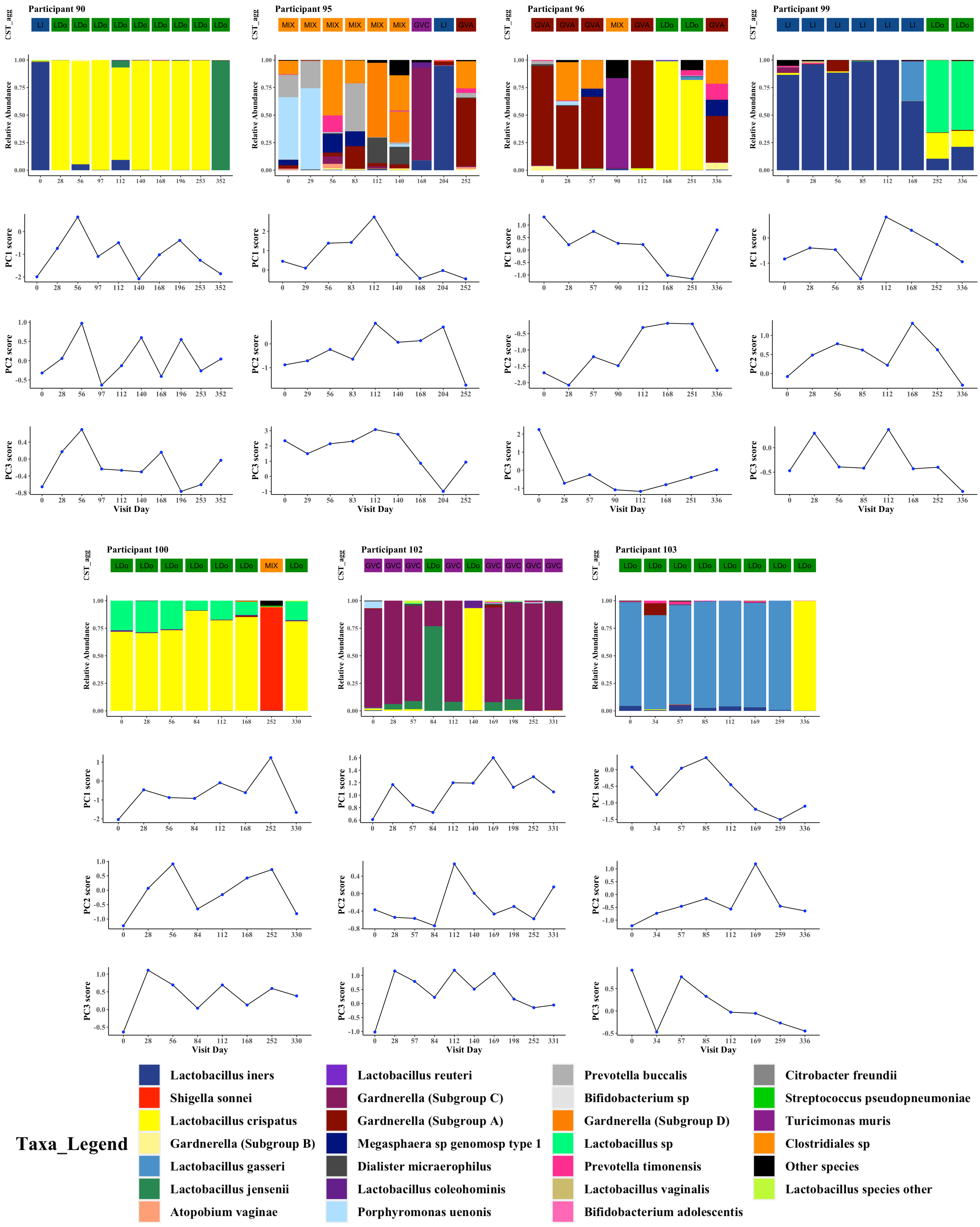

Participant 90
LI LDo LDo LDo LDo LDo LDo LDo LDo LDo
Participant 95
MIX MIX MIX MIX MIX MIX GVC LI GVA
Participant 96
GVA GVA GVA MIX GVA LDo LDo GVA
Participant 99
LI LI LI LI LI LI LDo LDo
Participant 100
LDo LDo LDo LDo LDo LDo MIX LDo
Participant 102
GVC GVC GVC LDo GVC LDo GVC GVC GVC GVC
Participant 103
LDo LDo LDo LDo LDo LDo LDo LDo
CST_agg
Relative Abundance
PC1 score
PC2 score
PC3 score
Visit Day
Taxa_Legend
Lactobacillus iners
Shigella sonnei
Lactobacillus crispatus
Gardnerella (Subgroup B)
Lactobacillus gasseri
Lactobacillus jensenii
Atopobium vaginae
Lactobacillus reuteri
Gardnerella (Subgroup C)
Gardnerella (Subgroup A)
Megasphaera sp genomosp type 1
Dialister micraerophilus
Lactobacillus coleohominis
Porphyromonas uenonis
Prevotella buccalis
Bifidobacterium sp
Gardnerella (Subgroup D)
Lactobacillus sp
Prevotella timonensis
Lactobacillus vaginalis
Bifidobacterium adolescentis
Citrobacter freundii
Streptococcus pseudopneumoniae
Turicimonas muris
Clostridiales sp
Other species
Lactobacillus species other

Appendix 6. Comparison of cervical T-cell subset cell counts among the different cervicovaginal microbial groupings. Log2-transformed cervical counts of (A) Ki67$^+$CD4$^+$T-cells, (B) CD38$^+$CD4$^+$T-cells and (C) HLA-DR$^+$CD4$^+$T-cells. Linear mixed model analysis of the association between specific cervicovaginal microbial communities (CST_agg) and log2-transformed cell counts of (D) Ki67$^+$CD4$^+$T-cells, (E) CD38$^+$CD4$^+$T-cells and (F) HLA-DR$^+$CD4$^+$T-cells. β-coefficients represent the mean change in log2-transformed cell count associated with a specific microbiome (CST_agg) when compared to Lactobacillus (non_iners) dominant (LDo) microbial communities. Blue symbols represent unadjusted models only utilizing microbial groupings as predictors. Red symbols represent models following multivariable adjustment for hormonal contraceptive type, HSV-II seropositivity at baseline, concomitant vulvovaginal candidiasis, age, use of BV antibiotics, use of other antibiotics, and use of antifungals prior to sample collection. The error bars in the raw cell count panels represent the standard error of the mean. The error bars from the linear mixed models represent the 95% confidence intervals associated with the estimates from the linear mixed models.

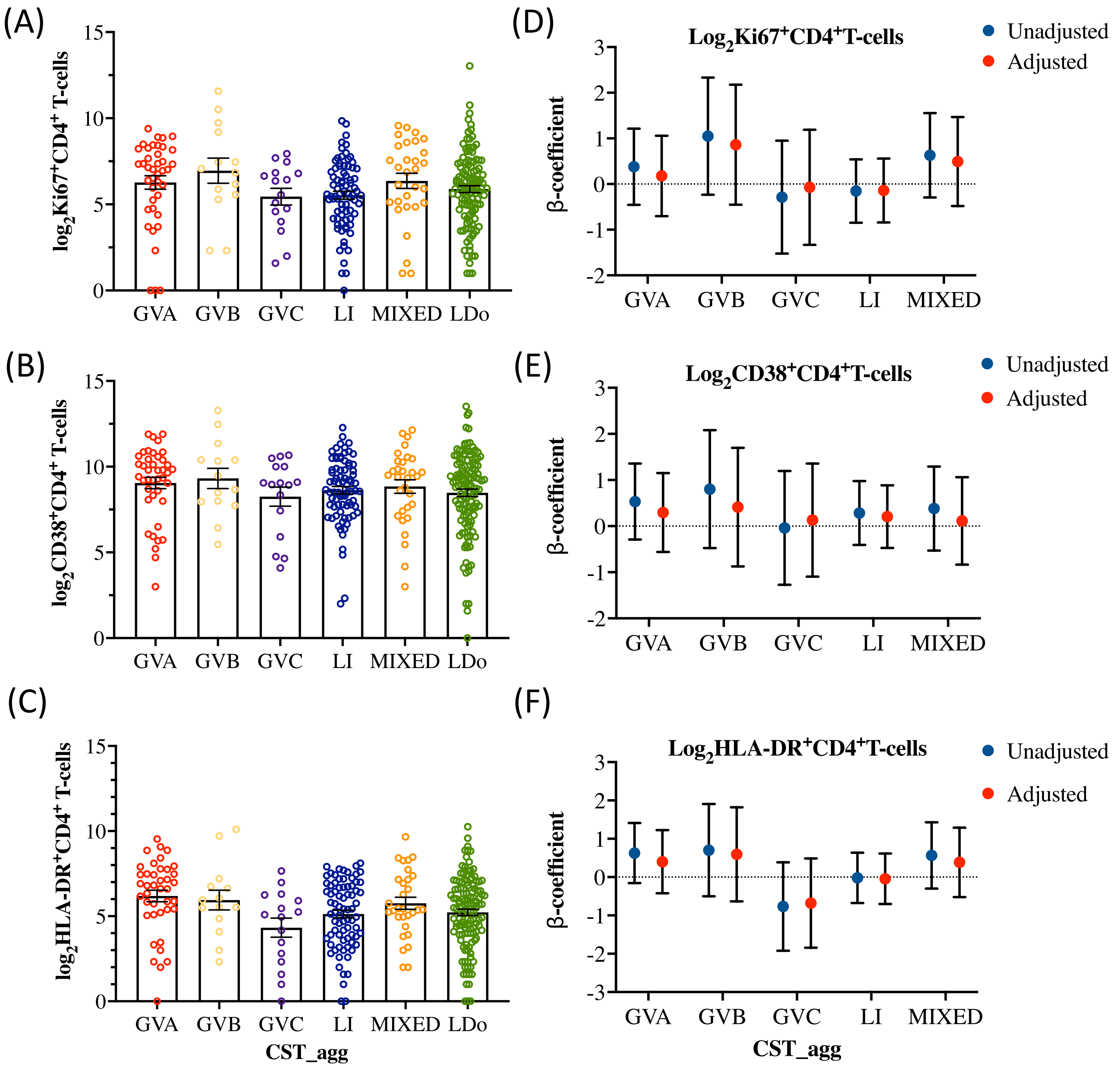

Appendix 7. Comparison of cervical integrin T-cell subset counts among the different cervicovaginal microbial groupings. Log2-transformed cervical counts of (A) $\alpha4^{+}\beta7^{hi}$ $CD4^{+}$T-cells (B) $\alpha4^{+}\beta1^{+}$ $CD4^{+}$T-cells (C) $\alpha E^{+}\beta7^{hi}$ $CD4^{+}$T-cells. Linear mixed model analysis of the association between specific cervicovaginal microbial communities (CST_agg) and log2-transformed cell counts of (D) $\alpha4^{+}\beta7^{hi}$ $CD4^{+}$T-cells (E) $\alpha4^{+}\beta1^{+}$ $CD4^{+}$T-cells (F) $\alpha E^{+}\beta7^{hi}$ $CD4^{+}$T-cells. β-coefficients represent the mean change in log2-transformed cell count associated with a specific microbiome (CST_agg) when compared to Lactobacillus (non_iners) dominant (LDo) microbial communities. Blue symbols represent unadjusted models only utilizing microbial groupings as predictors. Red symbols represent models following multivariable adjustment for hormonal contraceptive type, HSV-II seropositivity at baseline, concomitant vulvovaginal candidiasis, age, use of BV antibiotics, use of other antibiotics, and use of antifungals prior to sample collection. The error bars in the raw cell count panels represent the standard error of the mean. The error bars from the linear mixed models represent the 95% confidence intervals associated with the estimates from the linear mixed models.

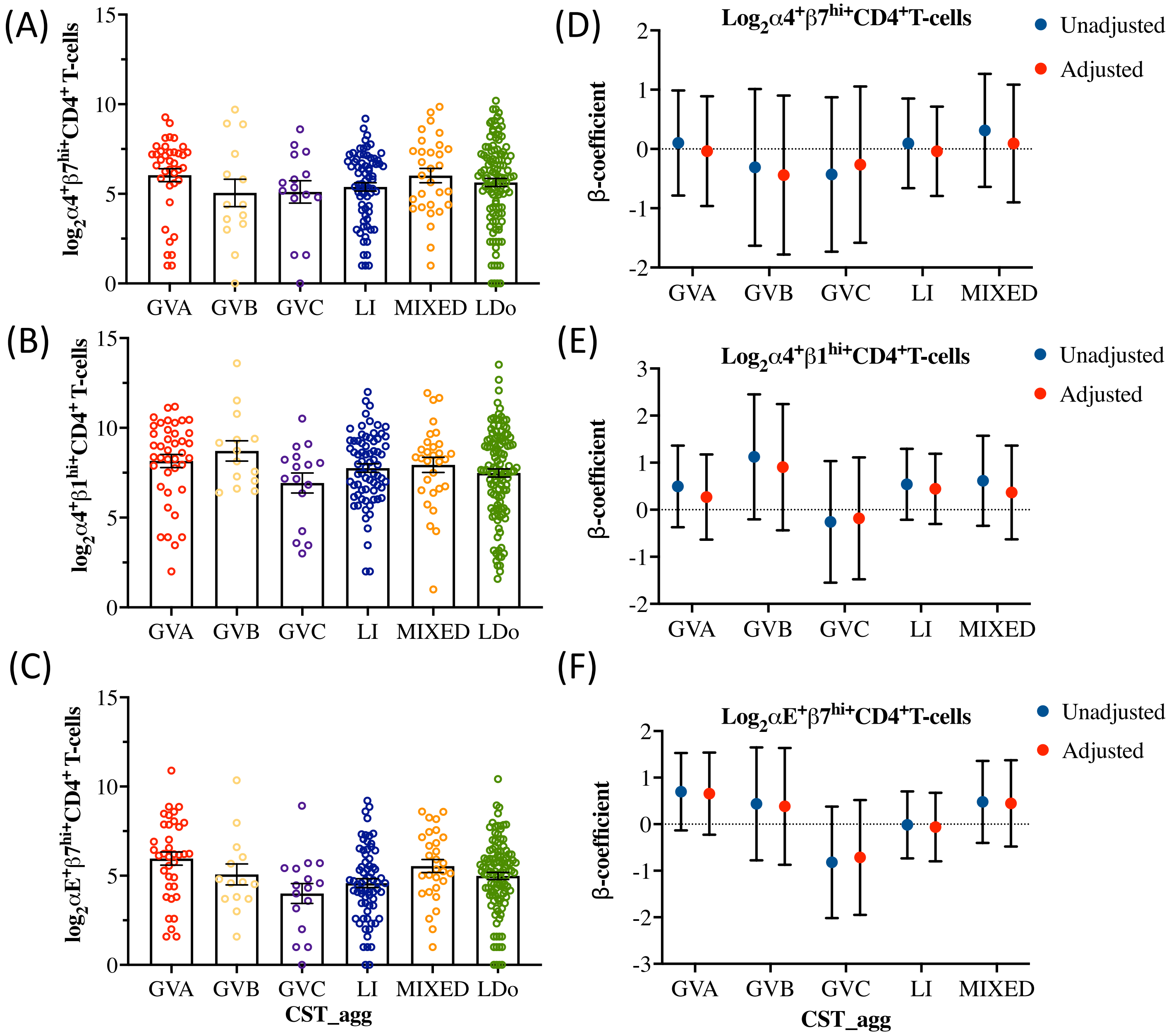

Appendix 8. Comparison of cellular marker expression on cervical T-lymphocytes among the different cervicovaginal microbial groupings. Raw relative frequencies of (A) CD38 and (B) HLA-DR expressed as percent of total $CD4^+$ T-cells. Linear mixed model analysis of the association between specific cervicovaginal microbial communities (CST_agg) and relative frequency of (C) CD38 and (D) HLA-DR expressed as percent of total $CD4^+$ T-cells. β-coefficients represent the mean change in relative frequency of a specific cellular marker associated with a specific microbiome (CST_agg) when compared to Lactobacillus (non_iners) dominant (LDo) microbial communities. Blue symbols represent unadjusted models only utilizing microbial groupings as predictors. Red symbols represent models following multivariable adjustment for hormonal contraceptive type, HSV-II seropositivity at baseline, concomitant vulvovaginal candidiasis, age, use of BV antibiotics, use of other antibiotics, and use of antifungals prior to sample collection. The error bars in the raw cell count panels represent the standard error of the mean. The error bars from the linear mixed models represent the 95% confidence intervals associated with the estimates from the linear mixed models.

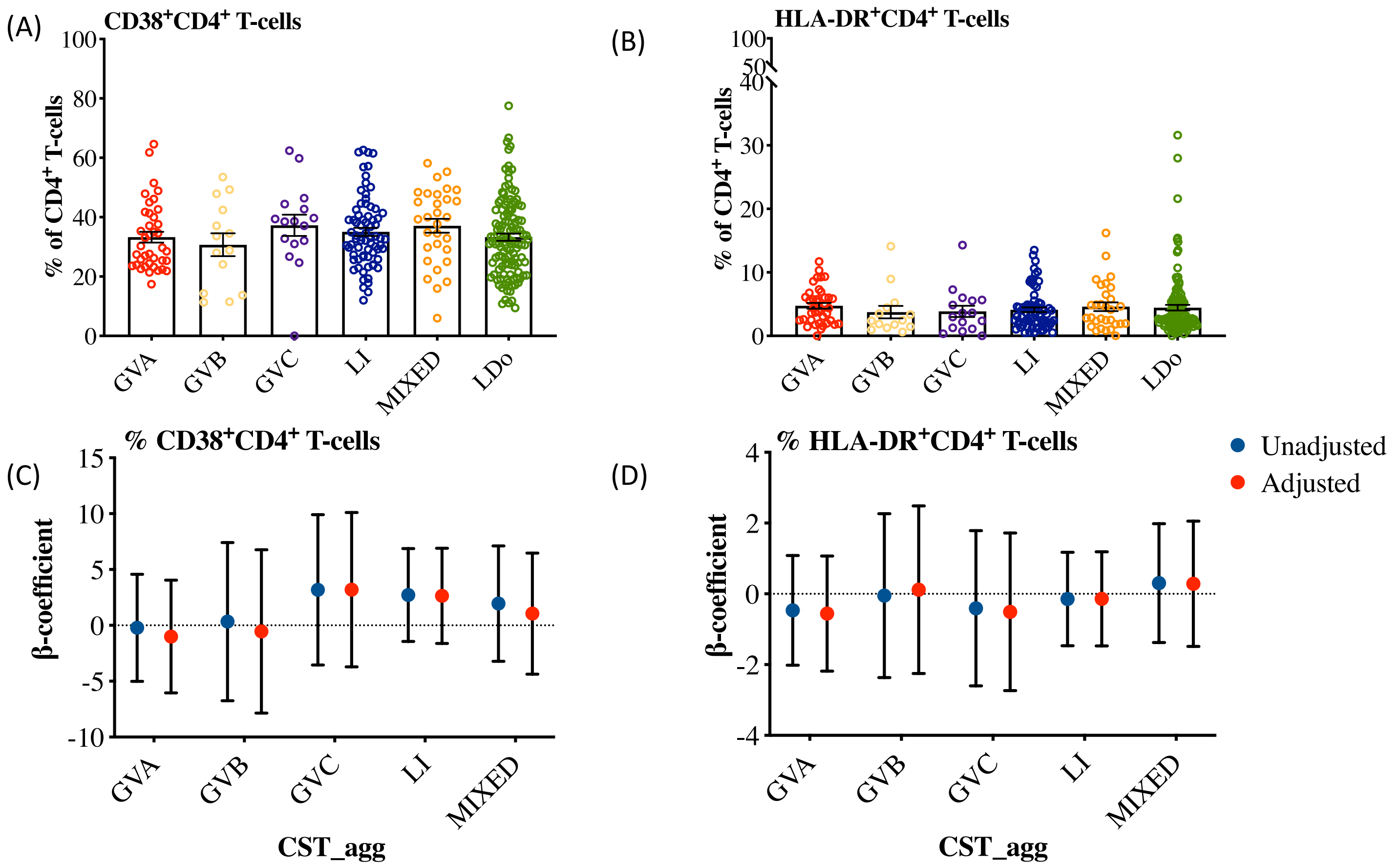

Supplement: Supplementary file 1 [file DataSheet_1.pdf]
